# Supplementary figures and images for: Associations of water contact frequency, duration, and activities with schistosome infection risk: A systematic review and meta-analysis
Source: PLoS Negl Trop Dis. 2023 Jun 14;17(6):e0011377. doi: 10.1371/journal.pntd.0011377 (PMC10266691; doi:10.1371/journal.pntd.0011377)

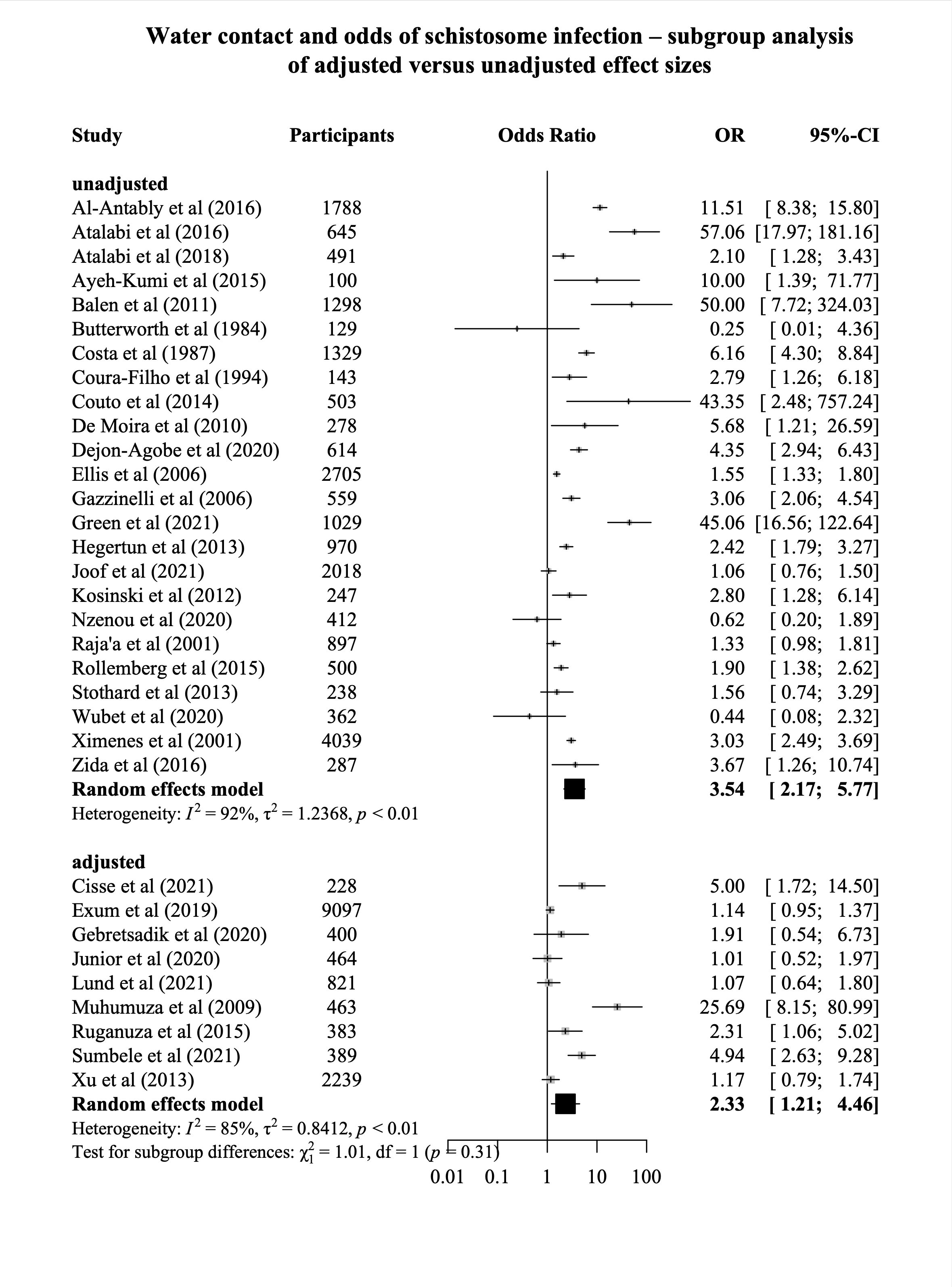

Supplement: S1 Fig — Full references of all included studies are available in S1 Table. (JPEG) [file pntd.0011377.s001.jpeg]

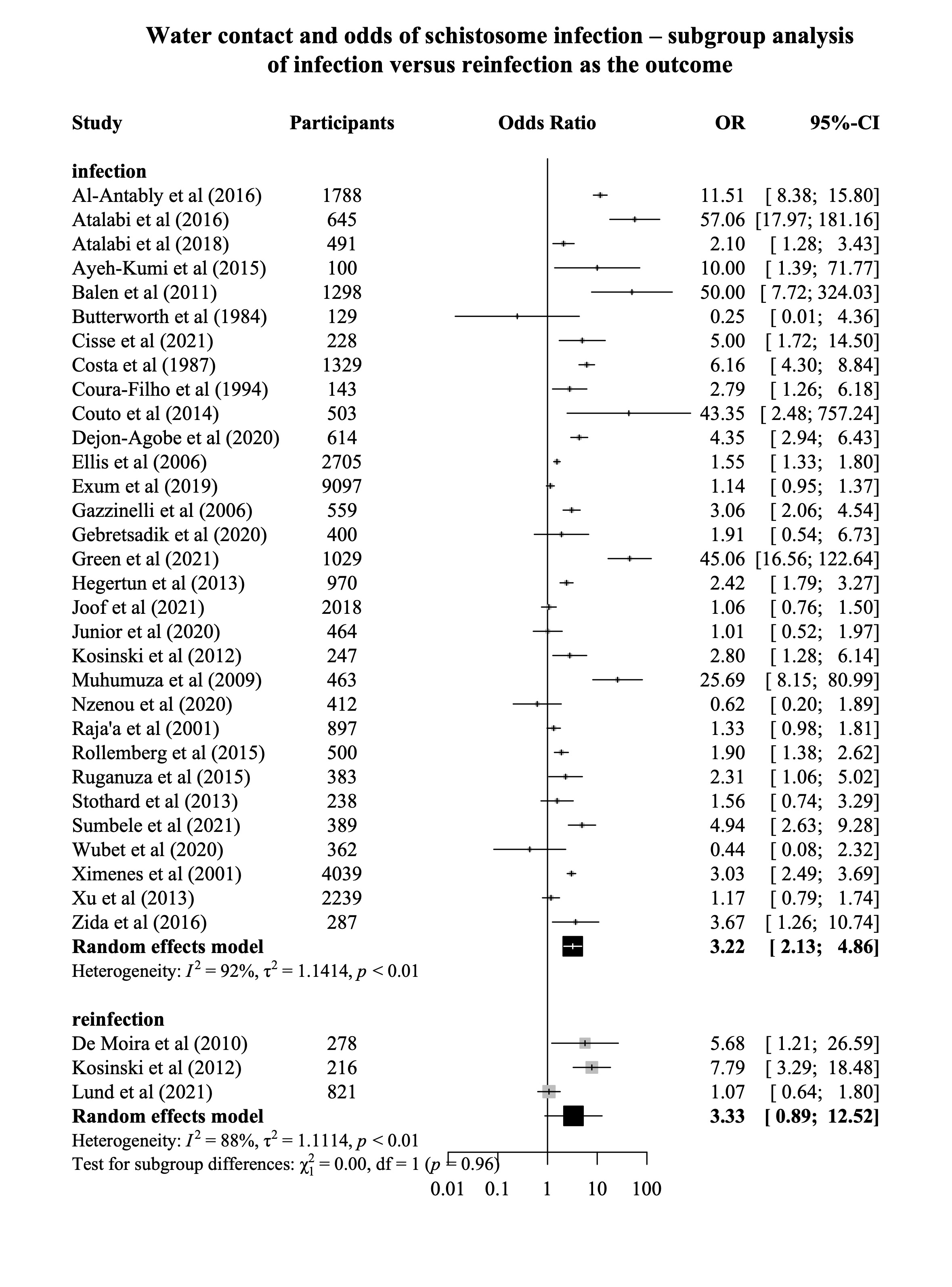

Supplement: S2 Fig — Full references of all included studies are available in S1 Table. (TIFF) [file pntd.0011377.s002.tiff]

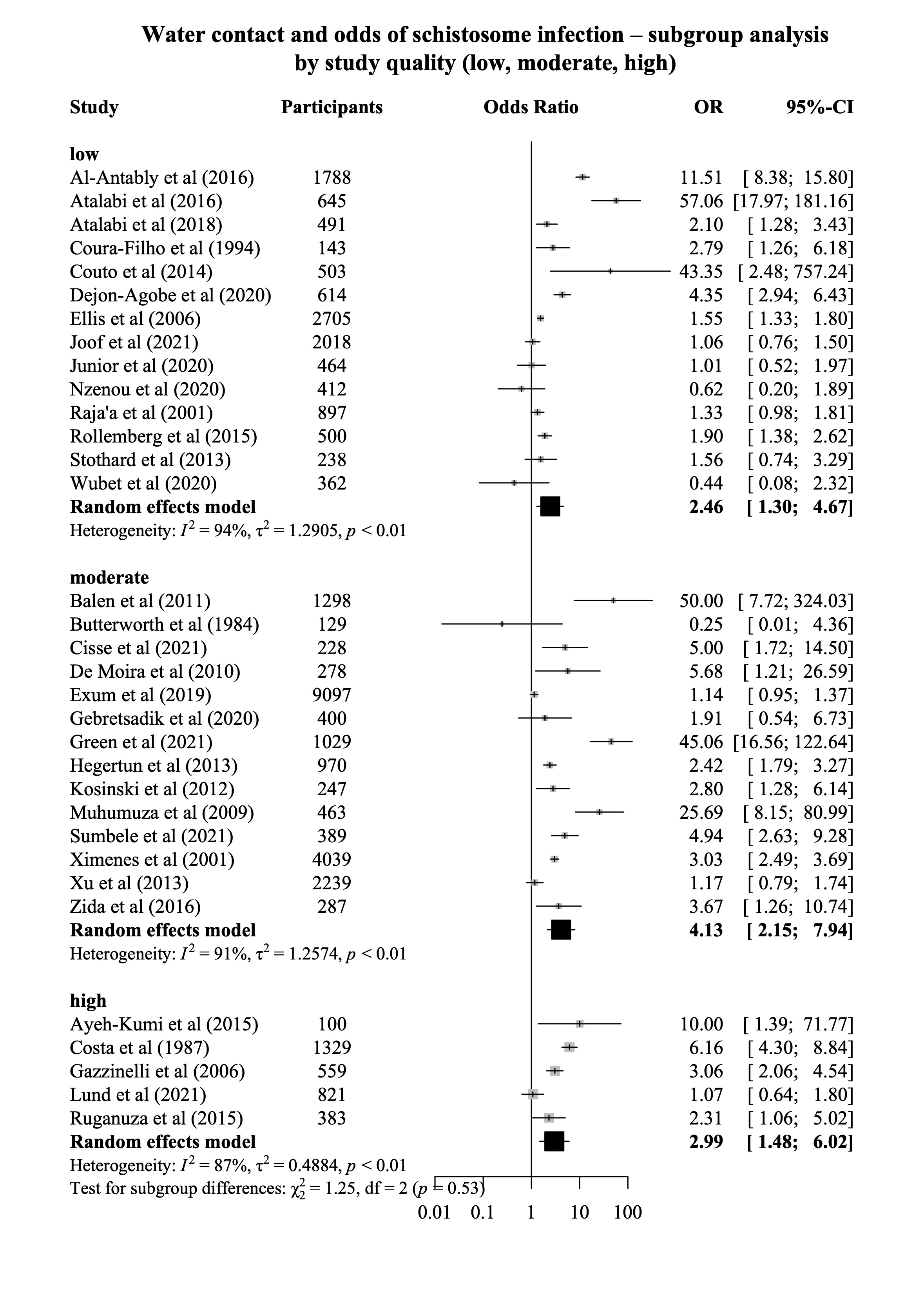

Supplement: S3 Fig — Full references of all included studies are available in S1 Table. (TIFF) [file pntd.0011377.s003.tiff]

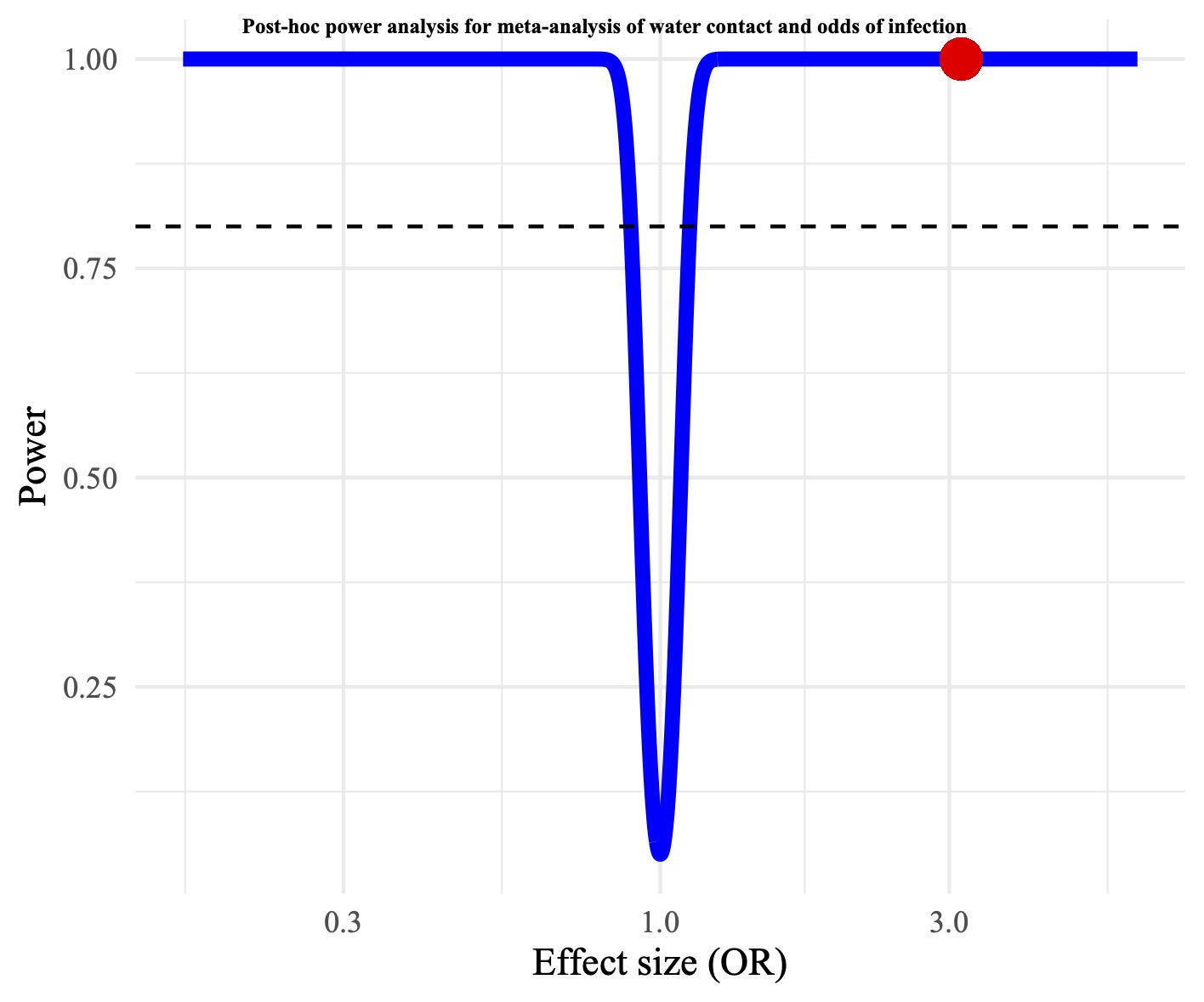

Supplement: S4 Fig — Post-hoc power calculation to detect an OR of 3.14 with 31 studies and a median study size of 503 participants >99% (parameters set to values from the meta-analysis). (TIFF) [file pntd.0011377.s004.tiff]

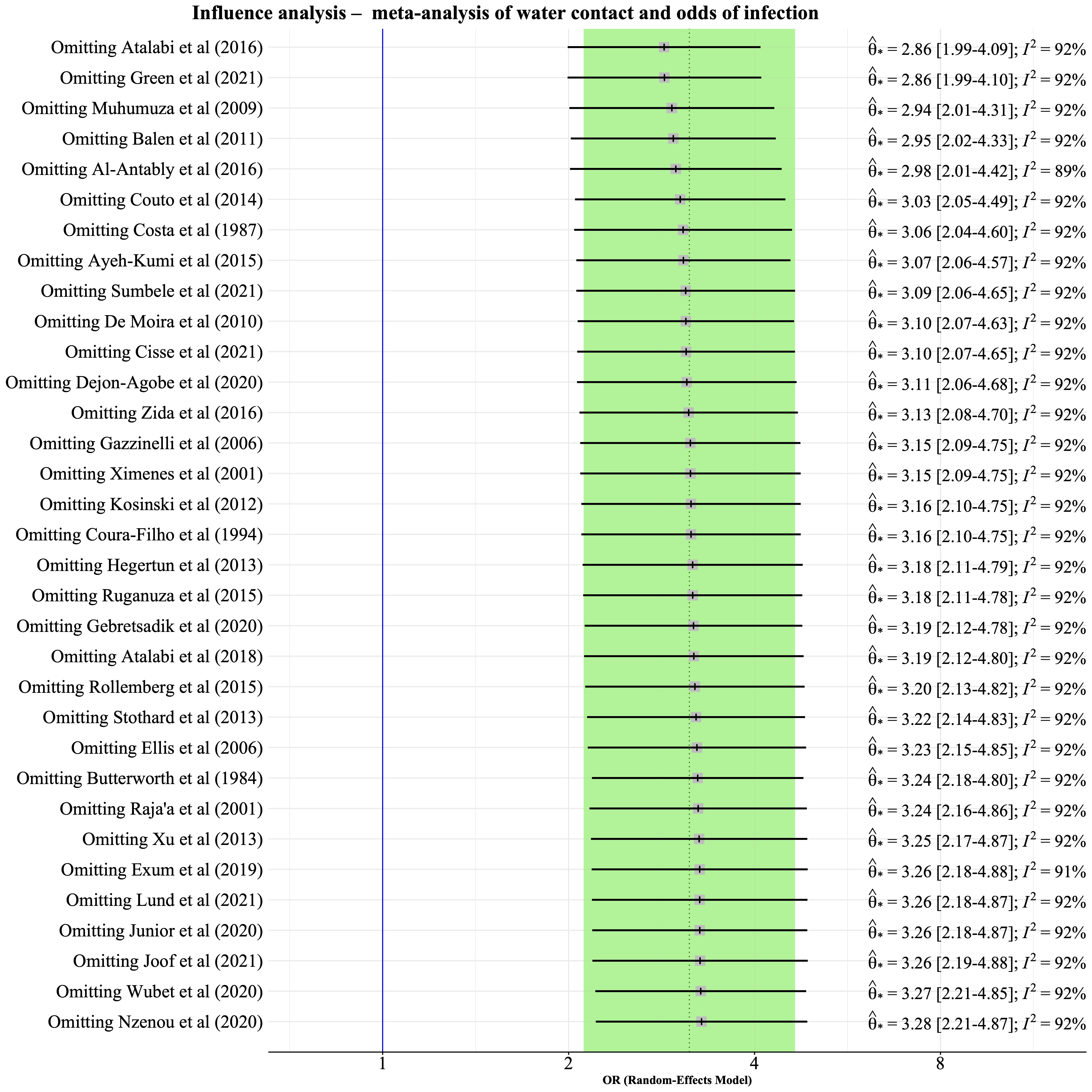

Supplement: S5 Fig — Full references of all included studies are available in S1 Table. (TIFF) [file pntd.0011377.s005.tiff]

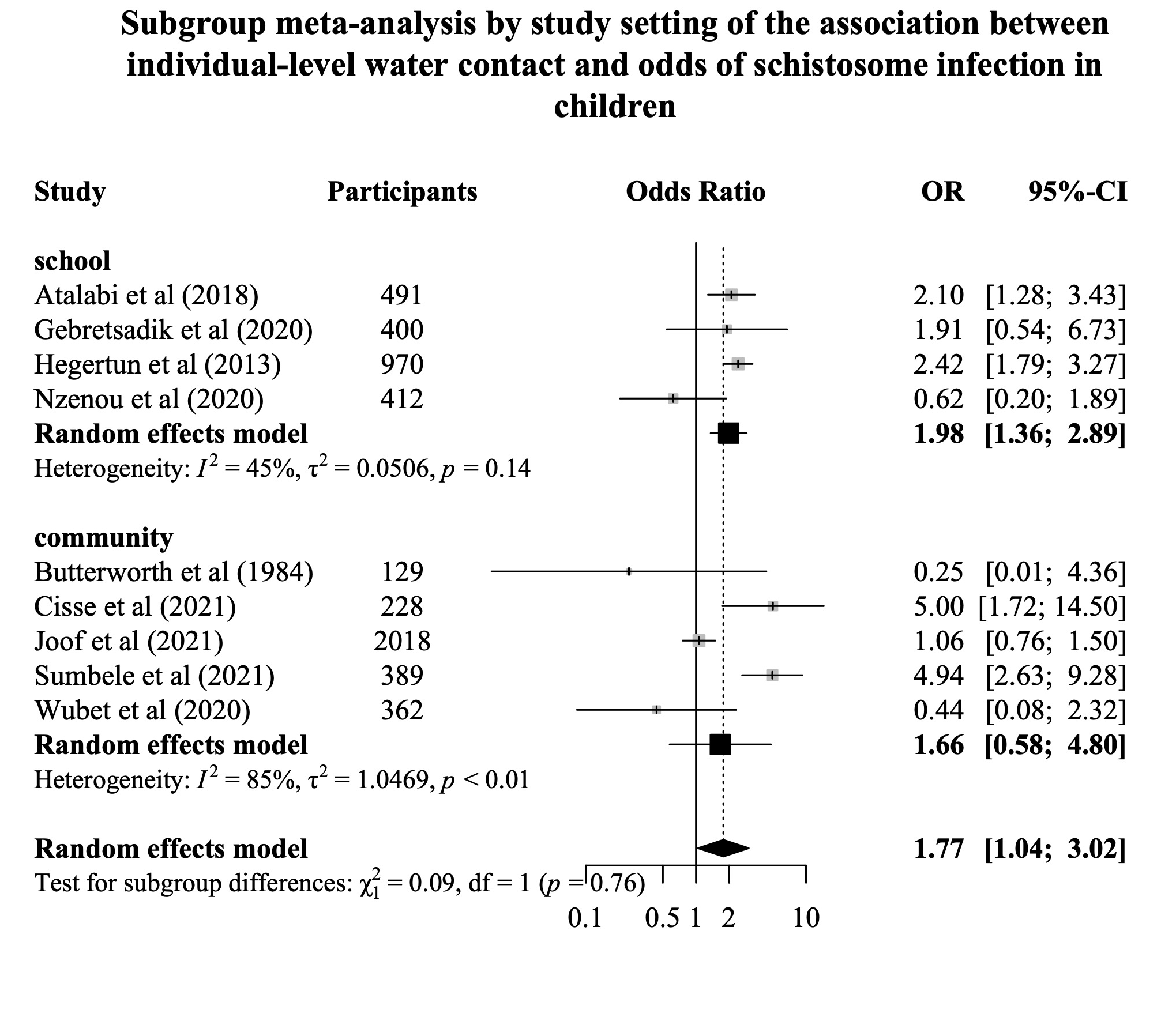

Supplement: S6 Fig — Full references of all included studies are available in S1 Table. (TIFF) [file pntd.0011377.s006.tiff]

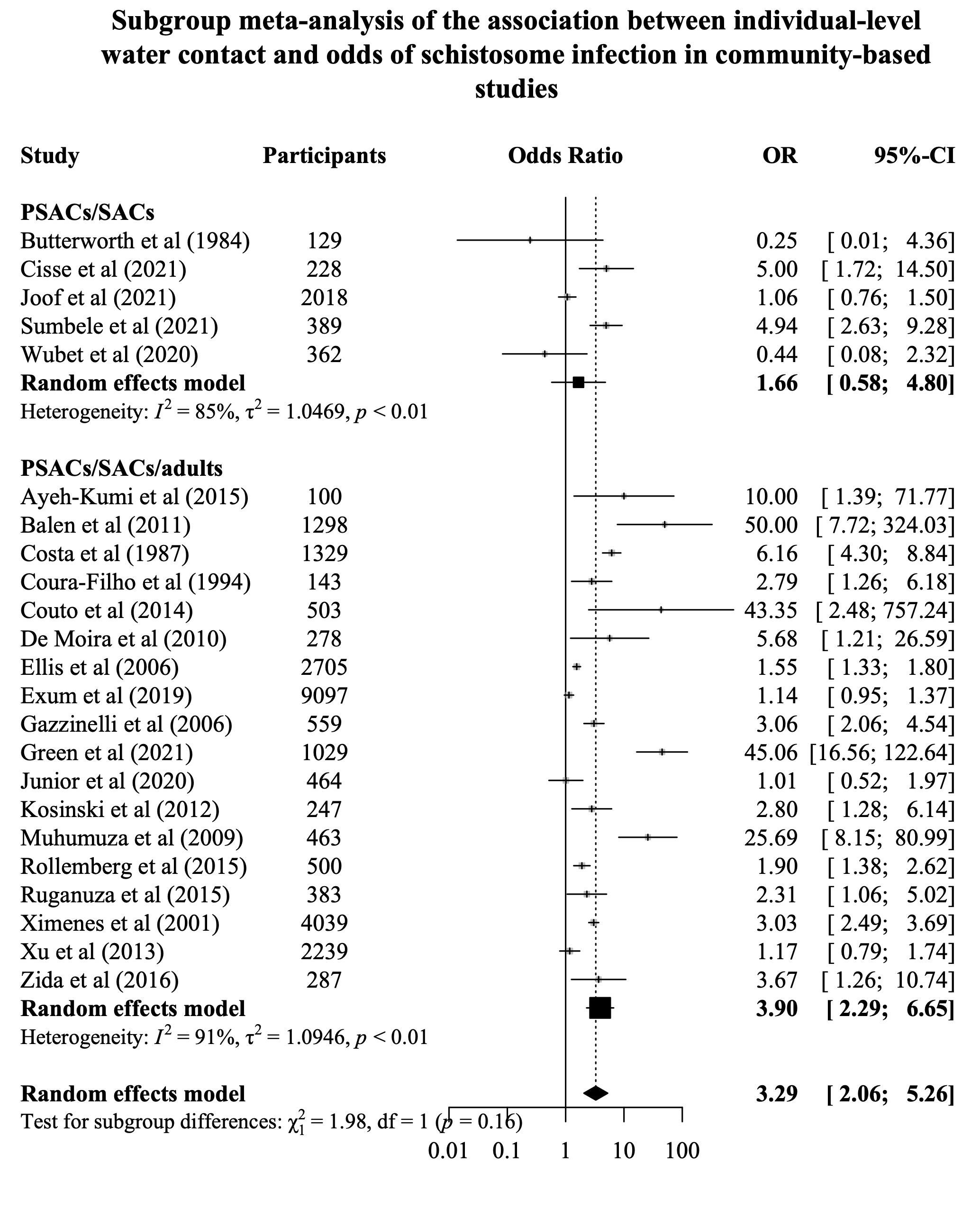

Supplement: S7 Fig — Full references of all included studies are available in S1 Table. (TIFF) [file pntd.0011377.s007.tiff]

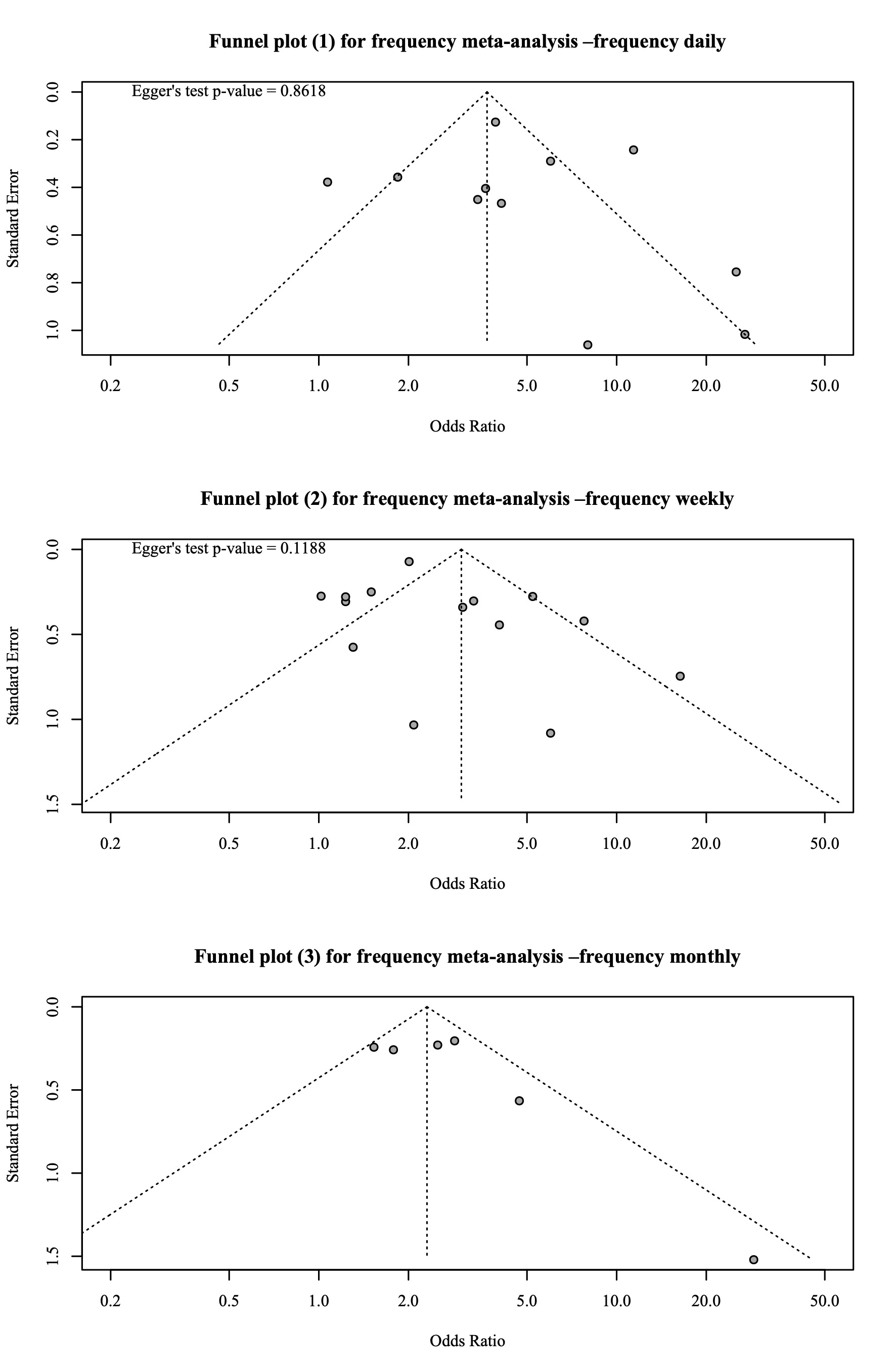

Supplement: S8 Fig — Egger’s test p-value reported for all categories with N≥10. (TIFF) [file pntd.0011377.s008.tiff]

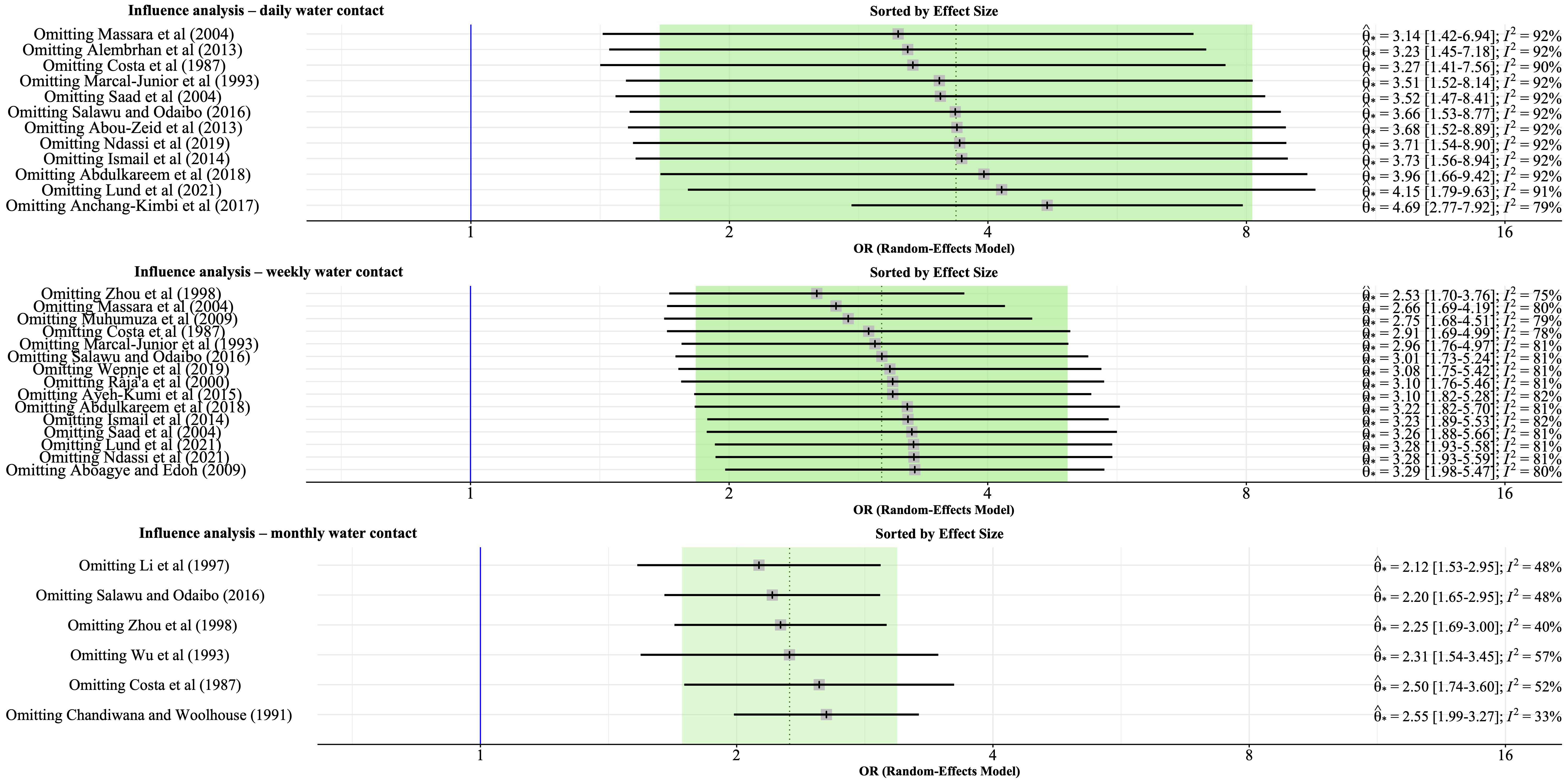

Supplement: S9 Fig — Full references of all included studies are available in S1 Table. (TIFF) [file pntd.0011377.s009.tiff]

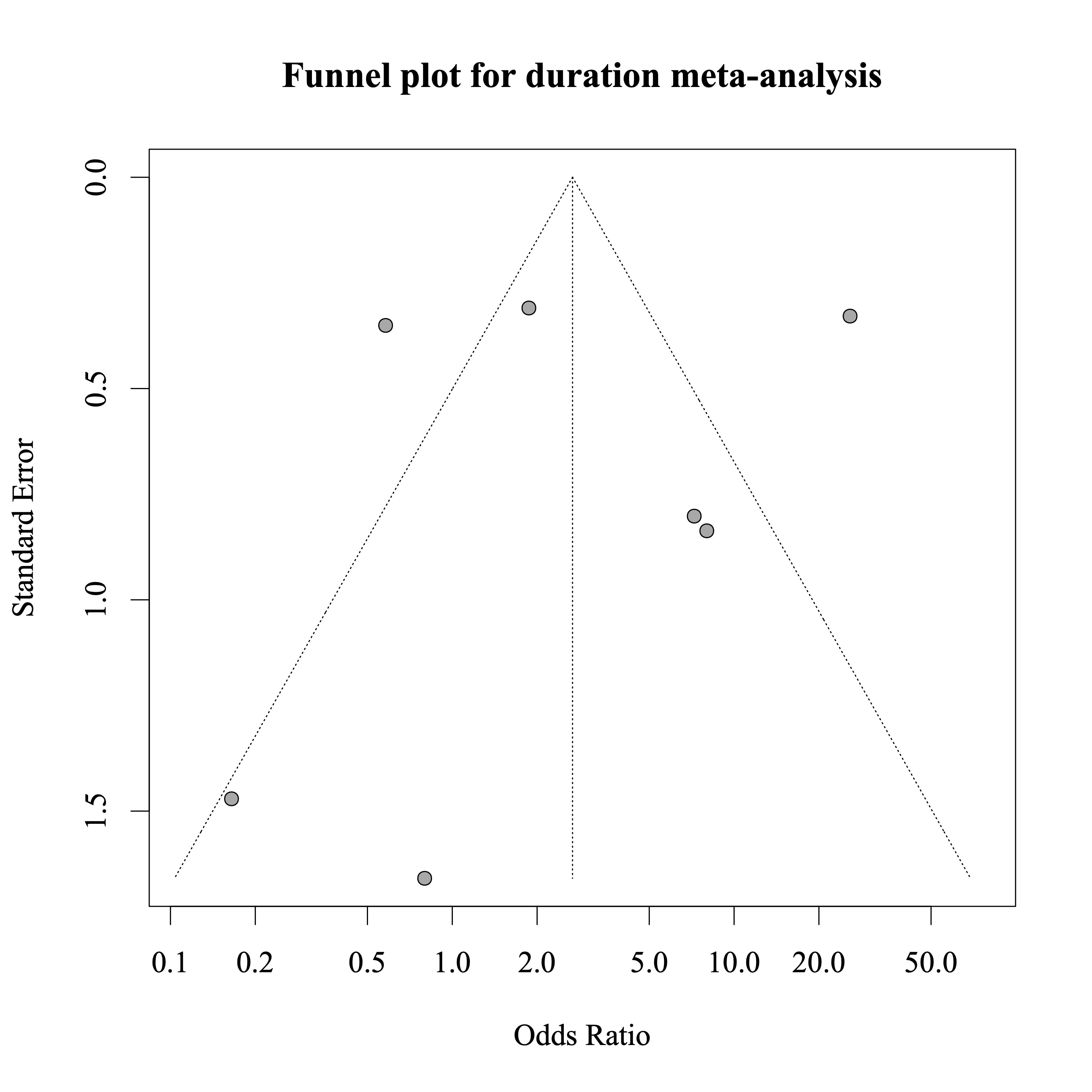

Supplement: S10 Fig — No Egger’s test was conducted due to the small number of studies (N = 6) because Egger’s test may lack statistical power to detect bias when N<10. (TIFF) [file pntd.0011377.s010.tiff]

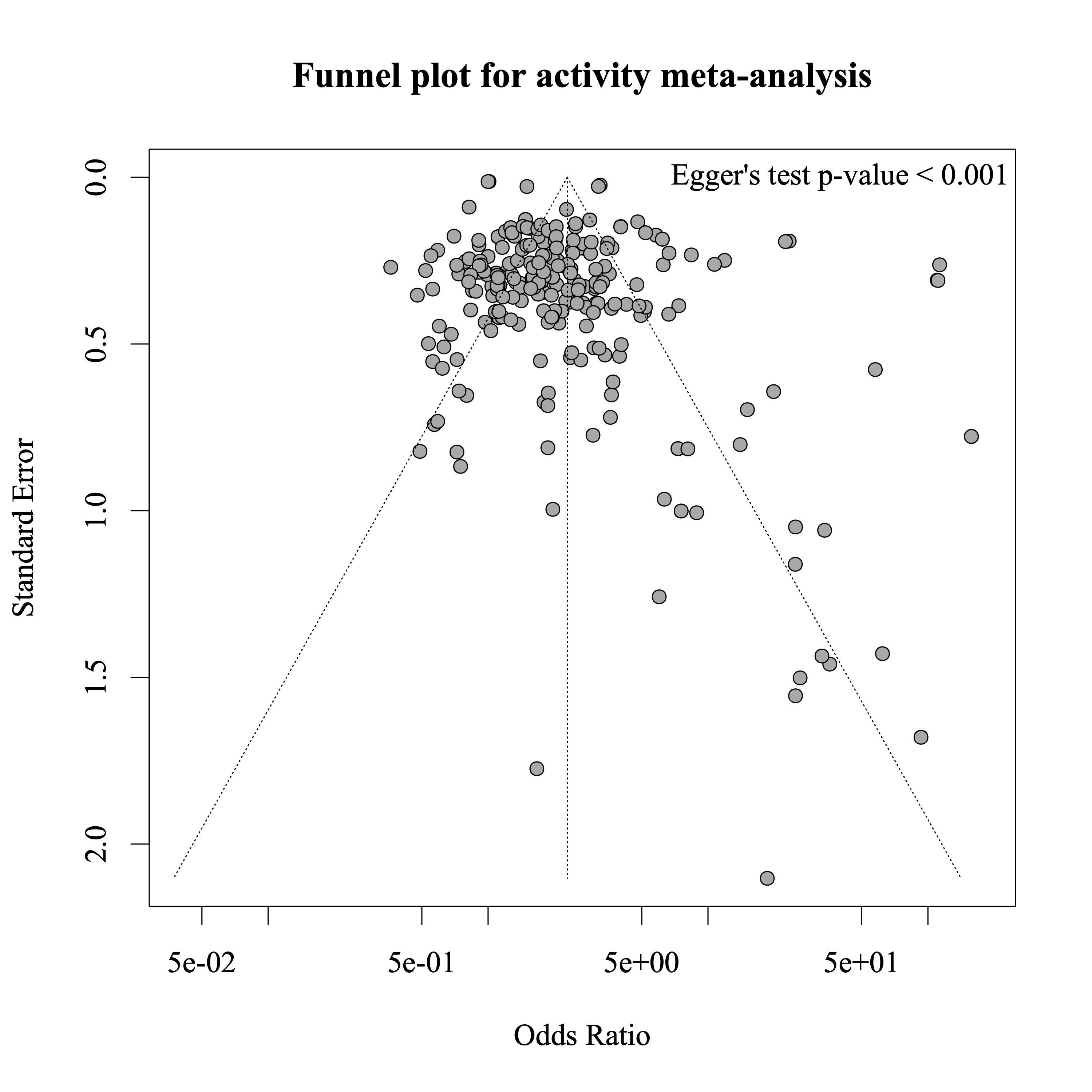

Supplement: S11 Fig — Egger’s test indicates significant funnel plot asymmetry (p<0.001). The funnel plot includes effect sizes and standard errors from all water contact activities reported across studies, using a multilevel model clustered by study. (TIFF) [file pntd.0011377.s011.tiff]
