# Supplementary material for: Associations of water contact frequency, duration, and activities with schistosome infection risk: A systematic review and meta-analysis
Source: PLoS Negl Trop Dis. 2023 Jun 14;17(6):e0011377. doi: 10.1371/journal.pntd.0011377 (PMC10266691; doi:10.1371/journal.pntd.0011377)
Supplement: S1 Table — (DOCX) [file pntd.0011377.s013.docx]

## **S1 Table. Study characteristics and full references**

| **Study** | **Country** | **Species** | **Locality** | **Setting** | **Study design** | **Population** | **Sample size** | **Water body** | **Tool** | **Exposure** |
| --- | --- | --- | --- | --- | --- | --- | --- | --- | --- | --- |
| Abdulkareem et al (2018) [1] | Nigeria | *Sh* | Rural | Community | Cross-sectional | SAC, older children and adults | 724 | River | Survey | Frequency, duration |
| Aboagye and Edoh (2009) [2] | Ghana | *Sh* | Peri-urban | Community | Cross-sectional | SAC, older children and adults | 309 | Lake | Survey | Frequency, activity |
| Abou-Zeid et al (2013) [3] | Sudan | *Sh* | Rural | School | Cross-sectional | SAC | 2302 | River | Survey | Frequency |
| Al-Antably et al (2016) [4] | Egypt | *Sh* | Urban, rural, peri-urban | Health clinic | Cross-sectional | SAC, older children and adults | 1788 | Canal | Survey | Any water contact |
| Al-Waleedi et al (2013) [5] | Yemen | *Sh* | Rural | Community | Cross-sectional | PSAC, SAC | 696 | Pond | Survey | Activity |
| Alebie et al (2014) [6] | Ethiopia | *Sm* | Urban | School | Cross-sectional | SAC, older children and adults | 384 | River, stream | Survey | Activity |
| Assefa et al (2013) [7] | Ethiopia | *Sh* | Peri-urban | School | Cross-sectional | SAC, older children and adults | 457 | River, dam | Survey | Frequency, activity |
| Amuta et al (2014) [8] | Nigeria | *Sh* | Rural | Health clinic | Cross-sectional | PSAC, SAC | 300 | River, stream, pond | Survey | Activity |
| Anchang-Kimbi et al (2017) [9] | Cameroon | *Sh* | Rural | Health clinic | Cross-sectional | SAC, older children and adults | 350 | Stream | Survey | Frequency |
| Angelo et al (2018) [10] | Tanzania | *Sh* | Rural | Community | Cohort | SAC | 250 | River, pond | Survey | Activity |
| Assaré et al (2020) [11] | Ivory Coast | *Sm, Sh* | Rural | School | Cohort | SAC | 274 | Pond, lake, stream | Survey | Activity |
| Atalabi et al (2018) [12] | Nigeria | *Sh* | Rural | School | Cross-sectional | SAC, older children and adults | 645 | Dam, pond, river, stream | Survey | Any water contact, activity |
| Atalabi et al (2016) [13] | Nigeria | *Sh* | Rural | School | Cross-sectional | SAC | 491 | River, pond, stream, lake | Survey | Any water contact, activity |
| Awoke et al (2013) [14] | Ethiopia | *Sh* | Rural | Community | Cross-sectional | SAC | 828 | Canal | Survey | Activity |
| Ayeh-Kumi et al (2015) [15] | Ghana | *Sh* | Rural | Community | Cross-sectional | PSAC, SAC, older children and adults | 171 | River | Survey | Any water contact, frequency, activity |
| Balen et al (2011) [16] | China | *Sm* | Rural | Community | Cross-sectional | PSAC, SAC, older children and adults | 1295 | River | Survey | Any water contact |
| Barreto (1991) [17] | Brazil | *Sm* | Peri-urban | Community | Cross-sectional | SAC | 1701 | Stream | Survey | Frequency, activity |
| Bekana et al (2021) [18] | Ethiopia | *Sm* | Rural | School | Cross-sectional | SAC | 798 | River, lake | Survey | Activity |
| Bolaji et al (2015) [19] | Nigeria | *Sh* | Rural | School | Cross-sectional | SAC, older children and adults | 150 | River | Survey | Activity, duration |
| Buchwald et al (2021) [20] | China | *Sj* | Rural | Community | Case-control | SAC, older children and adults | 603 | Pond, canal, field | Survey | Duration |
| Butterworth et al (1984) [21] | Kenya | *Sm* | Rural | Community | Cohort | PSAC | 129 | River | Direct water contact observation | Any water contact, duration |
| Campbell et al (2017) [22] | Cameroon | *Sh* | Rural | Community | Cross-sectional | PSAC, SAC, older children and adults | 338 | Lake | Survey | Activity |
| Chandiwana and Woolhouse (1991) [23] | Zimbabwe | *Sh* | Rural | Community | Cohort | PSAC, SAC, older children and adults | 465 | River | Direct water contact observation | Frequency |
| Chirundu et al (2007) [24] | Zimbabwe | *Sm, Sh* | Rural | School | Cross-sectional | SAC | 277 | River, stream | Survey | Activity |
| Cisse et al (2021) [25] | Burkina Faso | *Sh* | Rural | Community | Cross-sectional | PSAC | 230 | Pond | Survey | Any water contact, activity |
| Cooppan et al (1986) [26] | South Africa | *Sh* | Rural | School | Cross-sectional | SAC, older children and adults | 510 | River, stream, pond | Survey | Activity |
| Costa et al (1987) [27] | Brazil | *Sm* | Peri-urban | Community | Cross-sectional | PSAC, SAC, older children and adults | 1329 | Stream | Survey | Any water contact, frequency |
| Coura-Filho et al (1994) [28] | Brazil | *Sm* | Rural | Community | Cross-sectional | PSAC, SAC, older children and adults | 143 | Stream, field | Survey | Any water contact, activity |
| Couto et al (2014) [29] | Brazil | *Sm* | Urban, rural | Community | Cross-sectional | PSAC, SAC, older children and adults | 449 | River | Survey | Any water contact, activity |
| De Moira et al (2010) [30] | Uganda | *Sm* | Rural | Community | Cohort | SAC, older children and adults | 215 | Lake | Direct water contact observation | Any water contact |
| Dejenie et al (2013) [31] | Ethiopia | *Sm* | Rural | School | Cross-sectional | SAC, older children and adults | 500 | River, dam | Survey | Activity |
| Dejon-Agobe et al (2020) [32] | Gabon | *Sh* | Peri-urban | School | Cross-sectional | SAC, older children and adults | 614 | Stream | Survey | Any water contact |
| El-Khoby et al (2000) [33] | Egypt | *Sh* | Rural | Community | Cross-sectional | PSAC, SAC, older children and adults | 117230 | Canal | Survey | Activity |
| El-Sahn et al (2002) [34] | Egypt | *Sm* | Peri-urban | Community | Cross-sectional | PSAC, SAC, older children and adults | 552 | Canal | Survey | Activity |
| Ellis et al (2006) [35] | China | *Sm* | Rural | Community | Cross-sectional | SAC, older children and adults | 2705 | Lake | Survey | Any water contact |
| Enk et al (2010) [36] | Brazil | *Sm* | Rural | Community | Cross-sectional | PSAC, SAC, older children and adults | 1061 | Pond, swamp | Survey | Activity |
| Exum et al (2019) [37] | Uganda | *Sm* | Rural, peri-urban, urban | Community | Cross-sectional | PSAC, SAC, older children and adults | 9097 | Lake | Survey | Any water contact, activity |
| Fentahun et al (2021) [38] | Ethiopia | *Sm* | Rural | Community | Cross-sectional | SAC, older children and adults | 388 | Lake | Survey | Activity |
| Firmo et al (1996) [39] | Brazil | *Sm* | Peri-urban | Community | Case-control | PSAC, SAC, older children and adults | 916 | Stream, lake | Survey | Activity |
| Gazzinelli et al (2017) [40] | Brazil | *Sm* | Rural | Community | Cross-sectional | SAC, older children and adults | 559 | Stream, canal, dam | Survey | Any water contact |
| Gazzinelli et al (2006) [41] | Brazil | *Sm* | Rural, urban, peri-urban | School | Cohort | SAC | 506 | River, stream, canal, pond | Survey | Activity |
| Gebretsadik et al (2020) [42] | Ethiopia | *Sm* | Peri-urban | School | Cross-sectional | SAC | 400 | Unclear | Survey | Any water contact |
| Gebreyohanns et al (2018) [43] | Ethiopia | *Sm* | Urban | Community | Cross-sectional | SAC, older children and adults | 411 | River, stream, pond | Survey | Activity |
| Green et al (2021) [44] | Cameroon | *Sh* | Rural | Community | Cross-sectional | PSAC, SAC, older children and adults | 1029 | Stream | Survey | Any water contact, frequency, activity |
| Guimaraes et al (1985) [45] | Brazil | *Sm* | Rural | Community | Cross-sectional | PSAC, SAC, older children and adults | 686 | River | Survey | Frequency, activity |
| Hailu et al (2018) [46] | Ethiopia | *Sm* | Rural | School | Cross-sectional | SAC | 402 | River | Survey | Activity |
| Hammad et al (1996) [47] | Egypt | *Sm* | Rural | Community | Cross-sectional | SAC | 251 | Canal | Survey | Activity |
| Handzel et al (2003) [48] | Kenya | *Sm* | Rural | Community | Cross-sectional | SAC | 1246 | Lake | Survey | Frequency, activity |
| Hegertun et al (2013) [49] | South Africa | *Sh* | Rural | School | Cross-sectional | SAC | 970 | River, dam, lake, stream, pond | Survey | Any water contact |
| Houmsou et al (2010) [50] | Nigeria | *Sh* | Rural | Community | Cross-sectional | PSAC, SAC | 750 | River | Survey | Activity |
| Houmsou et al (2021) [51] | Nigeria | *Sh* | Rural | Community | Cross-sectional | PSAC, SAC | 1153 | Stream, river, pond | Survey | Activity |
| Houmsou et al (2016) [52] | Nigeria | *Sh* | Rural | Community | Cross-sectional | PSAC, SAC, older children and adults | 675 | River, dam | Survey | Activity |
| Ismail et al (2014) [53] | Sudan | *Sh* | Rural | School | Cross-sectional | SAC, older children and adults | 338 | River | Survey | Frequency, activity |
| Joof et al (2021) [54] | Gambia | *Sh* | Rural | Community | Cross-sectional | SAC | 2016 | River, stream, pond, rice paddy | Survey | Any water contact, activity |
| Junior et al (2020) [55] | Cameroon | *Sm, Sh* | Peri-urban, urban | Community | Cross-sectional | PSAC, SAC | 464 | River, stream | Survey | Any water contact |
| Keiser et al (2002) [56] | Ivory Coast | *Sm* | Rural | School | Cross-sectional | SAC | 325 | River | Survey | Activity |
| Kosinski et al (2012) [57] | Ghana | *Sh* | Rural | Community | Cohort | SAC, older children and adults | 117 | River | Direct water contact observation | Any water contact, duration |
| Kulinkina et al (2019) [58] | Ghana | *Sh* | Rural | Community | Cross-sectional | SAC | 897 | River, stream, pond | Survey | Activity |
| Li et al (1997) [59] | China | *Sj* | Rural | Community | Cross-sectional | PSAC, SAC, older children and adults | 1542 | Lake | Survey | Frequency |
| Lund et al (2021) [60] | Senegal | *Sh* | Rural | School, community | Cohort | SAC | 821 | River, lake | Survey | Any water contact, frequency |
| Mahmoud et al (1998) [61] | Egypt | *Sm* | Rural | Community | Cohort | SAC | 382 | Canal | Survey | Activity |
| Marcal-Junior et al (1993) [62] | Brazil | *Sm* | Rural | Community | Case-control | unclear | 192 | River | Survey | Frequency, activity |
| Masaku et al (2020) [63] | Kenya | *Sh* | Rural | School | Cross-sectional | PSAC, SAC | 653 | River, dam, lake | Survey | Activity |
| Massara et al (2004) [64] | Brazil | *Sh* | Peri-urban | School, community | Case-control | PSAC, SAC, older children and adults | 1061 | River, stream | Survey | Frequency, activity |
| Matthys et al (2007) [65] | Ivory Coast | *Sm* | Urban | Community | Cross-sectional | PSAC, SAC, older children and adults | 716 | River | Survey | Activity |
| Mengistu et al (2011) [66] | Ethiopia | *Sm* | Peri-urban | Community | Cross-sectional | PSAC, SAC, older children and adults | 517 | River | Survey | Activity |
| Mohammed et al (2018) [67] | Ethiopia | *Sm* | Rural | Community | Cross-sectional | SAC, older children and adults | 1011 | River | Survey | Activity |
| Morgas et al (2010) [68] | South Africa | *Sh* | Rural | School | Cross-sectional | SAC | 278 | River | Survey | Activity |
| Muhumuza et al (2009) [69] | Uganda | *Sm* | Rural | Community | Cross-sectional | SAC, older children and adults | 463 | Lake | Survey | Any water contact, frequency, duration |
| Munsami et al (2016) [70] | South Africa | *Sh* | Rural | School | Cross-sectional | SAC | 970 | River, dam, lake, stream, pond | Survey | Activity |
| Ndassi et al (2019) [71] | Cameroon | *Sh* | Rural | Community | Cross-sectional | PSAC, SAC, older children and adults | 1001 | Stream | Survey | Frequency |
| Ndassi et al (2021) [72] | Cameroon | *Sh* | Peri-urban | Community | Cross-sectional | SAC, older children and adults | 509 | Stream | Survey | Frequency |
| Ndukwe et al (2019) [73] | Nigeria | *Sh* | Rural, urban | Community | Cross-sectional | PSAC, SAC, older children and adults | 367 | River | Survey | Activity |
| Nigo et al (2021) [74] | Democratic Republic of the Congo | *Sm* | Rural | Community | Cross-sectional | PSAC, SAC, older children and adults | 707 | River, stream, lake | Survey | Activity |
| Njunda et al (2017) [75] | Cameroon | *Sh* | Rural | School | Cross-sectional | SAC | 382 | Dam | Survey | Frequency |
| Nzenou et al (2020) [76] | Cameroon | *Sm, Sh* | Rural | School | Cross-sectional | SAC | 412 | River, stream, swamp | Survey | Any water contact, activity |
| Olamiju et al (2022) [77] | Nigeria | *Sm, Sh* | Rural | Community | Cross-sectional | SAC, older children and adults | 432 | River | Survey | Activity |
| Olkeba et al (2022) [78] | Ethiopia | *Sm* | Rural | Community | Cross-sectional | older children and adults | 200 | River, lake | Survey | Activity |
| Phiri et al (2016) [79] | Malawi | *Sm* | Rural | Community | Randomised controlled trial | PSAC, SAC, older children and adults | 1642 | River | Survey | Activity |
| Raja'a et al (2001) [80] | Yemen | *Sm, Sh* | Rural | School | Cross-sectional | SAC, older children and adults | 230 | River | Survey | Frequency |
| Raja'a et al (2000) [81] | Yemen | *Sm, Sh* | Urban, rural | School | Cross-sectional | SAC, older children and adults | 897 | River | Survey | Any water contact |
| Rollemberg et al (2015) [82] | Brazil | *Sm* | Rural | Community | Cross-sectional | PSAC, SAC, older children and adults | 500 | Canal | Survey | Any water contact |
| Rudge et al (2008) [83] | Tanzania | *Sh* | Rural | School | Cross-sectional | SAC | 150 | Stream, pond | Survey | Activity |
| Ruganuza et al (2015) [84] | Tanzania | *Sm* | Rural | Community | Cross-sectional | PSAC, SAC | 400 | Lake | Survey | Any water contact |
| Saad et al (2004) [85] | Egypt | *Sm* | Rural, urban | Community | Cross-sectional | PSAC, SAC, older children and adults | 1409 | River | Survey | Frequency, activity, duration |
| Salawu and Odaibo (2016) [86] | Nigeria | *Sh* | Rural | Health clinic | Cross-sectional | SAC, older children and adults | 237 | River | Survey | Frequency, activity |
| Stothard et al (2013) [87] | Tanzania | *Sh* | Rural | School | Cross-sectional | SAC, older children and adults | 238 | Stream, pond | Survey | Activity |
| Sumbele et al (2021) [88] | Cameroon | *Sh* | Peri-urban | Community | Cross-sectional | SAC | 389 | Stream | Survey | Any water contact |
| Takeuchi et al (2019) [89] | Kenya | *Sm* | Rural | School | Cross-sectional | SAC | 424 | Lake | Survey | Activity |
| Umar et al (2017) [90] | Nigeria | *Sh* | Rural | School | Cross-sectional | SAC | 325 | River, stream, pond | Survey | Activity |
| Utzinger et al (2000) [91] | Ivory Coast | *Sm* | Rural | Community | Cross-sectional | SAC | 322 | River | Survey | Activity |
| Wagatsuma et al (2003) [92] | Ghana | *Sh* | Rural | School | Cross-sectional | SAC, older children and adults | 354 | River, stream, pond | Survey | Activity |
| Wanjala et al (2013) [93] | Kenya | *Sm* | Rural | School | Cross-sectional | SAC | 972 | Lake | Survey | Activity |
| Wepnje et al (2019) [94] | Cameroon | *Sh* | Rural | Health clinic | Cross-sectional | SAC, older children and adults | 368 | Stream | Survey | Frequency |
| Wu et al (1993) [95] | China | *Sj* | Rural | Community | Cohort | PSAC, SAC, older children and adults | 740 | Lake | Survey | Frequency |
| Wubet et al (2020) [96] | Ethiopia | *Sm* | Rural | Community | Cross-sectional | SAC | 362 | River | Survey | Any water contact, activity |
| Ximenes et al (2001) [97] | Brazil | *Sm* | Urban | Community | Cross-sectional | SAC, older children and adults | 2015 | Stream, river | Survey | Any water contact, activity |
| Xu et al (2013) [98] | China | *Sj* | Rural | Community | Cross-sectional | SAC, older children and adults | 2339 | River | Survey | Any water contact |
| Yacoub and Southgate (1987) [99] | Iraq | *Sh* | Rural | Community | Cross-sectional | PSAC, SAC, older children and adults | 209 | River | Survey | Activity |
| Zhou et al (1998) [100] | Ivory Coast | *Sj* | Rural | Community | Cross-sectional | SAC | 478 | Lake | Survey | Frequency, activity |
| Zida et al (2016) [101] | Burkina Faso | *Sh* | Rural | Community | Cross-sectional | SAC, older children and adults | 287 | Dam | Survey | Any water contact, activity |

PSAC = pre-school age children

SAC = school-age children

*Sh* = *Schistosoma haematobium*

*Sm* = *Schistosoma mansoni*

*Sj* = *Schistosoma japonicum*

**References**

1. Abdulkareem B, Habeeb K, Kazeem A, Adam A, Samuel U. Urogenital Schistosomiasis among Schoolchildren and the Associated Risk Factors in Selected Rural Communities of Kwara State, Nigeria. J Trop Med. 2018;2018. doi:10.1155/2018/6913918

2. Aboagye IF, Edoh D. Investigation of the risk of infection of urinary schistosomiasis at Mahem and Galilea communities in the Greater Accra Region of Ghana. West Afr J Appl Ecol. 2009;15: 27–32.

3. Abou-Zeid A, Abkar T, Mohamed R. Schistosomiasis infection among primary school students in a war zone, Southern Kordofan State, Sudan: a cross-sectional study. BMC Public Health. 2013;13. doi:10.1186/1471-2458-13-643

4. Al-Antably ASA, Fouad SA, Basyoni MMA, Hassan MA. Socio-demographic risk factors of schistosomiasis mansoni in patients with gastrointestinal symptoms: a seroprevalence study in Egypt. J Egypt Soc Parasitol. 2016;46: 367–374.

5. Al-Waleedi AA, El-Nimr NA, Hasab AA, Bassiouny HK, Al-Shibani LA. Urinary schistosomiasis among schoolchildren in Yemen: prevalence, risk factors, and the effect of a chemotherapeutic intervention. J Egypt Public Health Assoc. 2013;88: 130–6. doi:10.1097/01.EPX.0000441277.96615.96

6. Getachew Alebie, Berhanu Erko, Mulugeta Aemero, Beyene Petros. Epidemiological study on *Schistosoma mansoni* infection in Sanja area, Amhara region, Ethiopia. Parasit Vectors. 2014;7.

7. Alembrhan Assefa, Tadesse Dejenie, Zewdneh Tomass. Infection prevalence of *Schistosoma mansoni* and associated risk factors among schoolchildren in suburbs of Mekelle city, Tigray, Northern Ethiopia. Momona Ethiop J Sci. 2013;5: 174–188.

8. Amuta E, Houmsou R. Prevalence, intensity of infection and risk factors of urinary schistosomiasis in pre- school and school aged children in Guma Local Government Area, Nigeria. Asian Pac J Trop Dis. 2014;7: 34–39. doi:10.1016/S1995-7645(13)60188-1

9. Anchang-Kimbi JK, Elad DM, Sotoing GT, Achidi EA. Coinfection with *Schistosoma haematobium* and *Plasmodium falciparum* and anaemia severity among pregnant women in Munyenge, Mount Cameroon Area: a cross-sectional study. J Parasitol Res. 2017;2017: Article-6173465. doi:10.1155/2017/6173465

10. Angelo T, Buza J, Kinung’hi SM, Kariuki HC, Mwanga JR, Munisi DZ, et al. Geographical and behavioral risks associated with *Schistosoma haematobium* infection in an area of complex transmission. Parasit Vectors. 2018;11: 481. doi:10.1186/s13071-018-3064-5

11. Assare R, N’Tamon R, Bellai L, Koffi J, Mathieu T, Ouattara M, et al. Characteristics of persistent hotspots of *Schistosoma mansoni* in western Cote d’Ivoire. Parasit Vectors. 2020;13. doi:10.1186/s13071-020-04188-x

12. Atalabi T, Adoh S, Eze K. The current epidemiological status of urogenital schistosomiasis among primary school pupils in Katsina State, Nigeria: An imperative for a scale up of water and sanitation initiative and mass administration of medicines with Praziquantel. PLoS Negl Trop Dis. 2018;12. doi:10.1371/journal.pntd.0006636

13. Atalabi TE, Lawal U, Akinluyi FO. Urogenital schistosomiasis and associated determinant factors among senior high school students in the Dutsin-Ma and Safana Local Government Areas of Katsina State, Nigeria. Infect Dis Poverty. 2016;5.

14. Worku Awoke, Melkamu Bedimo, Molalign Tarekegn. Prevalence of schistosomiasis and associated factors among students attending at elementary schools in Amibera District, Ethiopia. Open J Prev Med. 2013;3: 199–204. doi:10.4236/ojpm.2013.32027

15. Ayeh-Kumi PF, Obeng-Nkrumah N, Baidoo D, Teye J, Asmah RH. High levels of urinary schistosomiasis among children in Bunuso, a rural community in Ghana: an urgent call for increased surveillance and control programs. J Parasit Dis. 2015;39: 613–623. doi:10.1007/s12639-013-0411-5

16. Balen J, Raso G, Li Y-S, Zhao Z-Y, Yuan L-P, Williams GM, et al. Risk factors for helminth infections in a rural and a peri-urban setting of the Dongting Lake area, People’s Republic of China. Int J Parasitol. 2011;41: 1165–1173. doi:10.1016/j.ijpara.2011.07.006

17. Barreto ML. Geographical and socioeconomic factors relating to the distribution of *Schistosoma mansoni* infection in an urban area of north-east Brazil. Bull World Health Organ. 1991;69: 93–102.

18. Teshome Bekana, Nega Berhe, Tadesse Eguale, Mulugeta Aemero, Girmay Medhin, Begna Tulu, et al. Prevalence and factors associated with intestinal schistosomiasis and human fascioliasis among school children in Amhara regional state, Ethiopia. Trop Med Health. 2021;49. doi:10.1186/s41182-021-00326-y

19. Bolaji OS, Elkanah FA, Ojo JA, Ojurongbe O, Adeyeba OA. Prevalence and intensity of *Schistosoma haematobium* among school children in Ajase-Ipo, Kwara State, Nigeria. Asian J Biomed Pharm Sci. 2015;5: 6–11.

20. Buchwald AG, Grover E, Van Dyke J, Kechris K, Lu D, Liu Y, et al. Human Mobility Associated With Risk of *Schistosoma japonicum* Infection in Sichuan, China. Am J Epidemiol. 2021;190: 1243–1252. doi:10.1093/aje/kwaa292

21. Butterworth AE, Dalton PR, Dunne DW, Mugambi M, Ouma JH, Richardson BA, et al. Immunity after treatment of human schistosomiasis mansoni. I. Study design, pretreatment observations and the results of treatment. Trans R Soc Trop Med Hyg. 1984;78: 108–123. doi:10.1016/0035-9203(84)90190-1

22. Campbell SJ, Stothard JR, O’Halloran F, Sankey D, Durant T, Ombede DE, et al. Urogenital schistosomiasis and soil-transmitted helminthiasis (STH) in Cameroon: An epidemiological update at Barombi Mbo and Barombi Kotto crater lakes assessing prospects for intensified control interventions. Infect Dis Poverty. 2017;6: 49. doi:10.1186/s40249-017-0264-8

23. Chandiwana SK, Woolhouse ME. Heterogeneities in water contact patterns and the epidemiology of *Schistosoma haematobium*. Parasitology. 1991;103 Pt 3: 363–370. doi:10.1017/s0031182000059874

24. Chirundu D, Chimusoro A, Jones D, Midzi N, Mabaera B, Apollo T, et al. Schistosomiasis infection among school children in the Zhaugwe resettlement area, Zimbabwe April 2005. Cent Afr J Med. 2007;53: 6–11.

25. Cisse M, Sangare I, Djibougou AD, Tahita MC, Gnissi S, Bassinga JKW, et al. Prevalence and risk factors of Schistosoma mansoni infection among preschool-aged children from Panamasso village, Burkina Faso. Parasit Vectors. 2021;14: 185. doi:10.1186/s13071-021-04692-8

26. Cooppan RM, Schutte CH, Mayet FG, Dingle CE, Van Deventer JM, Mosese PG. Morbidity from urinary schistosomiasis in relation to intensity of infection in the Natal Province of South Africa. Am J Trop Med Hyg. 1986;35: 765–76.

27. Costa MFFL, Magalhaes MHA, Rocha RS, Antunes CMF, Katz N. Water-contact patterns and socioeconomic variables in the epidemiology of schistosomiasis mansoni in an endemic area in Brazil. Bull World Health Organ. 1987;65: 57–66.

28. Coura-Filho P, Rocha RS, Farah MW, Da Silva GC, Katz N. Identification of factors and groups at risk of infection with *Schistosoma mansoni*: a strategy for the implementation of control measures? Rev Inst Med Trop Sao Paulo. 1994;36: 245–253. doi:10.1590/S0036-46651994000300009

29. Couto L, Tibirica S, Pinheiro I, Mitterofhe A, Lima A, Castro M, et al. Neglected tropical diseases: prevalence and risk factors for schistosomiasis and soil-transmitted helminthiasis in a region of Minas Gerais State, Brazil. Trans R Soc Trop Med Hyg. 2014;108: 363–371. doi:10.1093/trstmh/tru054

30. Moira AP de, Fulford AJC, Kabatereine NB, Ouma JH, Booth M, Dunne DW. Analysis of complex patterns of human exposure and immunity to schistosomiasis mansoni: the influence of age, sex, ethnicity and IgE. PLoS Negl Trop Dis. 2010;4: e820. doi:10.1371/journal.pntd.0000820

31. Tadesse Dejenie, Kabeta Legese, Zewdneh Tomas, Solomon Kiros. Index of potential contamination for intestinal schistosomiasis among school children of Raya Alamata District, Northern Ethiopia. Momona Ethiop J Sci. 2013;5: 32–48.

32. Dejon-Agobe J, Honkpehedji Y, Zinsou J, Edoa J, Adegbite B, Mangaboula A, et al. Epidemiology of Schistosomiasis and Soil-Transmitted Helminth Coinfections among Schoolchildren Living in Lambarene, Gabon. Am J Trop Med Hyg. 2020;103: 325–333. doi:10.4269/ajtmh.19-0835

33. El-Khoby T, Galal N, Fenwick A, Barakat R, El-Hawey A, Nooman Z, et al. The epidemiology of schistosomiasis in Egypt: Summary findings in nine governorates. Am J Trop Med Hyg. 2000;62: 88–99. doi:10.4269/ajtmh.2000.62.88

34. Amel A. ES, Abdel Ghanny M. EM, Amany I. S, Hanan F. I. Current status of *S. mansoni* infection in El-prince village, Alexandria governorate. J Egypt Public Health Assoc. 2002;77: 537–52.

35. Ellis M, Li Y, Rong Z, Chen H, McManus D. Familial aggregation of human infection with *Schistosoma japonicum* in the Poyang Lake region, China. Int J Parasitol. 2006;36: 71–77. doi:10.1016/j.ijpara.2005.09.006

36. Enk M, Lima A, Barros H, Massara C, Coelho P, Schall V. Factors related to transmission of and infection with *Schistosoma mansoni* in a village in the South-eastern Region of Brazil. Mem Inst Oswaldo Cruz. 2010;105: 570–577. doi:10.1590/S0074-02762010000400037

37. Exum NG, Kibira SPS, Ssenyonga R, Nobili J, Shannon AK, Ssempebwa JC, et al. The prevalence of schistosomiasis in Uganda: A nationally representative population estimate to inform control programs and water and sanitation interventions. Akullian A, editor. PLoS Negl Trop Dis. 2019;13: e0007617. doi:10.1371/journal.pntd.0007617

38. Fentahun A, Hailu T, Alemu G. Prevalence of Intestinal Parasites and *Schistosoma mansoni* and Associated Factors among Fishermen at Lake Tana, Northwest Ethiopia. BioMed Res Int. 2021;2021: 4534689. doi:10.1155/2021/4534689

39. Firmo JLOA, Costa MFLE, Guerra HL, Rocha RS. Urban Schistosomiasis: Morbidity, Sociodemographic Characteristics and Water Contact Patterns Predictive of Infection. Int J Epidemiol. 1996;25: 1292–1300. doi:10.1093/ije/25.6.1292

40. Gazzinelli A, Oliveira-Prado R, Matoso L, Veloso B, Andrade G, Kloos H, et al. *Schistosoma mansoni* reinfection: Analysis of risk factors by classification and regression tree (CART) modeling. PLoS One. 2017;12. doi:10.1371/journal.pone.0182197

41. Gazzinelli A, Velasquez-Melendez G, Crawford S, LoVerde P, Correa-Oliveira R, Kloos H. Socioeconomic determinants of schistosomiasis in a poor rural area in Brazil. ACTA Trop. 2006;99: 260–271. doi:10.1016/j.actatropica.2006.09.001

42. Gebretsadik D, Tesfaye M, Adamu A, Zewde G. Prevalence of Intestinal Parasitic Infection and Its Associated Factors Among School Children in Two Primary Schools in Harbu Town, North East Ethiopia: Cross-Sectional Study. Pediatr Health Med Ther. 2020;11: 179–188. doi:10.2147/PHMT.S252061

43. Alganesh Gebreyohanns, Melese Hailu Legese, Mistire Wolde, Gemechu Leta, Geremew Tasew. Prevalence of intestinal parasites versus knowledge, attitude and practices (KAPs) with special emphasis to *Schistosoma mansoni* among individuals who have river water contact in Addiremets town, Western Tigray, Ethiopia. PLoS ONE. 2018;13: e0204259. doi:10.1371/journal.pone.0204259

44. Green A, Anchang-Kimbi J, Wepnje G, Ndassi V, Kimbi H. Distribution and factors associated with urogenital schistosomiasis in the Tiko Health District, a semi-urban setting, South West Region, Cameroon. Infect Dis Poverty. 2021;10. doi:10.1186/s40249-021-00827-2

45. Guimaraes D.C., De Barros H.L., Katz N. A clinical epidemiologic study in a schistosomiasis mansoni endemic area. Rev Inst Med Trop Sao Paulo. 1985;27: 123–131. doi:10.1590/S0036-46651985000300003

46. Hailu T, Alemu M, Abera B, Mulu W, Yizengaw E, Genanew A, et al. Multivariate analysis of factors associated with *Schistosoma mansoni* and hookworm infection among primary school children in rural Bahir Dar, Northwest Ethiopia. Trop Dis Travel Med Vaccines. 2018;4: 4. doi:10.1186/s40794-018-0064-6

47. Hammad T, AbdelWahab M, DeClaris N, ElSahly A, ElKady N, Strickland G. Comparative evaluation of the use of artificial neural networks for modelling the epidemiology of schistosomiasis mansoni. Trans R Soc Trop Med Hyg. 1996;90: 372–376. doi:10.1016/S0035-9203(96)90509-X

48. Handzel T, Karanja DMS, Addiss DG, Hightower AW, Rosen DH, Colley DG, et al. Geographic distribution of schistosomiasis and soil-transmitted helminths in Western Kenya: implications for anthelminthic mass treatment. Am J Trop Med Hyg. 2003;69: 318–323.

49. Hegertun I, Gundersen K, Kleppa E, Zulu S, Gundersen S, Taylor M, et al. S-haematobium as a Common Cause of Genital Morbidity in Girls: A Cross-sectional Study of Children in South Africa. PLoS Negl Trop Dis. 2013;7. doi:10.1371/journal.pntd.0002104

50. Houmsou R.S., Kela S.L., Suleiman M.M., Ogidi J.A. Perceptions and assessment of risk factors in *Schistosoma haematobium* infection in Buruku and Katsina-Ala Local Government Areas of Benue State-Nigeria. Internet J Infect Dis. 2010;8.

51. Houmsou RS, Wama EB, Agere H, Uniga JA, Bingbeng JB, Jerry JT, et al. Integrated control of river and pond water as an exposure source to urogenital schistosomiasis of rural inhabitants in southern Taraba State, Nigeria. J Water Sanit Hyg Dev. 2021;11: 546–557. doi:10.2166/washdev.2021.257

52. Houmsou RS, Agere H, Wama BE, Bingbeng JB, Amuta EU, Kela SL. Urinary schistosomiasis among children in Murbai and Surbai communities of Ardo-Kola Local Government Area, Taraba State, Nigeria. J Trop Med. 2016;2016: Article-9831265.

53. Ismail H, Hong S, Babiker A, Hassan R, Sulaiman M, Jeong H, et al. Prevalence, risk factors, and clinical manifestations of schistosomiasis among school children in the White Nile River basin, Sudan. Parasit Vectors. 2014;7. doi:10.1186/s13071-014-0478-6

54. Joof E, Sanyang A, Camara Y, Sey A, Baldeh I, Jah S, et al. Prevalence and risk factors of schistosomiasis among primary school children in four selected regions of The Gambia. PLoS Negl Trop Dis. 2021;15. doi:10.1371/journal.pntd.0009380

55. Junior EE, Eyong EM, Dilonga HM, Patrick IV, Nicholas T. Prevalence, Infection Intensity and Risk Factors of Schistosomiasis and Soil Transmitted Helminthiasis among School Aged Children in Tiko Health District, Southwest Cameroon: A Community-Based Cross-Sectional Study. Int J Trop Dis Health. 2020; 12–29. doi:10.9734/ijtdh/2020/v41i730292

56. Keiser J, N’Goran E, Singer B, Lengeler C, Tanner M, Utzinger J. Association between *Schistosoma mansoni* and hookworm infections among schoolchildren in Cote d’Ivoire. Acta Trop. 2002;84: 31–41. doi:10.1016/S0001-706X(02)00135-3

57. Kosinski K, Adjei M, Bosompem K, Crocker J, Durant J, Osabutey D, et al. Effective Control of *Schistosoma haematobium* Infection in a Ghanaian Community following Installation of a Water Recreation Area. PLoS Negl Trop Dis. 2012;6. doi:10.1371/journal.pntd.0001709

58. Kulinkina A, Kosinski K, Adjei M, Osabutey D, Gyamfi B, Biritwum N, et al. Contextualizing Schistosoma haematobium transmission in Ghana: Assessment of diagnostic techniques and individual and community water-related risk factors. Acta Trop. 2019;194: 195–203. doi:10.1016/j.actatropica.2019.03.016

59. Li YS, Ross AGP, Yu DB, Li Y, Williams GM, McManus DP. An evaluation of *Schistosoma japonicum* infections in three villages in the Dongting lake region of China: I. Prevalence, intensity and morbidity before the implementation of adequate control strategies. Acta Trop. 1998;68: 77–91. doi:10.1016/S0001-706X(97)00077-6

60. Lund AJ, Sokolow SH, Jones IJ, Wood CL, Ali S, Chamberlin A, et al. Exposure, hazard, and vulnerability and their contribution to *Schistosoma haematobium* re-infection in northern Senegal. Lancet Planet Health. 2021;5: S10. doi:10.1016/S2542-5196(21)00094-2

61. Mahmoud M O, Maha T, Phillip J. Ma. Pattern and psycho-social, behavioral environmental risk factors for schistosomiasis infection among Egyptian school children. New Egypt J Med. 1998;18: 227–236.

62. Marçal Júnior O, Hotta LK, Patucci RM de J, Glasser CM, Dias LC de S. Schistosomiasis mansoni in an area of low transmission. II. Risk factors for infection. Rev Inst Med Trop Säo Paulo. 1993;35: 331–335.

63. Masaku J, Njomo DW, Njoka A, Okoyo C, Mutungi FM, Njenga SM. Soil-transmitted helminths and schistosomiasis among pre-school age children in a rural setting of Busia County, Western Kenya: a cross-sectional study of prevalence, and associated exposures. BMC Public Health. 2020;20. doi:10.1186/s12889-020-08485-z

64. Massara C, Peixoto S, Barros H, Enk M, Carvalho O, Schall V. Factors associated with schistosomiasis mansoni in a population from the municipality of jaboticatubas, state of Minas Gerais, Brazil. Mem Inst Oswaldo Cruz. 2004;99: 127–134. doi:10.1590/S0074-02762004000900023

65. Matthys B, Tschannen A, Tian-Bi N, Comoe H, Diabate S, Traore M, et al. Risk factors for *Schistosoma mansoni* and hookworm in urban farming communities in western Cote d’Ivoire. Trop Med Int Health. 2007;12: 709–723. doi:10.1111/j.1365-3156.2007.01841.x

66. Hailu H, Kassa T, Mengistu M, Shimelis T, Terefe A, Torben W. Human Intestinal Schistosomiasis in Communities Living Near three Rivers of Jimma Town; South Western Ethiopia. Ethiop J Health Sci. 2011;21: 111–118.

67. Mohammed J, Weldegebreal F, Teklemariam Z, Mitiku H. Clinico-epidemiology, malacology and community awareness of Schistosoma mansoni in Haradenaba and Dertoramis kebeles in Bedeno district, eastern Ethiopia. SAGE Open Med. 2018;6: 2050312118786748. doi:10.1177/2050312118786748

68. Gundersen SG, Kjetland EF, Kvalsvig JD, Taylor M, Thomassen Morgas DE. Schistosomiasis and Water-Related Practices in School Girls in Rural KwaZulu-Natal; South Africa. Afr J Infect Online. 2010;25: 30–33.

69. Muhumuza S, Kitimbo G, Oryema-Lalobo M, Nuwaha F. Association between socio economic status and schistosomiasis infection in Jinja District, Uganda. Trop Med Int Health. 2009;14: 612–619. doi:10.1111/j.1365-3156.2009.02273.x

70. Munsami A, Mitchell C, Lachenicht L, Kvalsvig JD, Kjetland EF, Taylor M. The role of socio-cultural-cognition in disease prevalence and risky behaviour among children: a conceptual framework. J AIDS Clin Res. 2016;7: 631.

71. Ndassi VD, Anchang-Kimbi JK, Sumbele IUN, Wepnje GB, Kimbi HK. Prevalence and risk factors associated with S. haematobium egg excretion during the dry season, six months following mass distribution of praziquantel (PZQ) in 2017 in the Bafia Health Area, south west region Cameroon: a cross-sectional study. J Parasitol Res. 2019;2019. doi:10.1155/2019/4397263

72. Ndassi V, Anchang-Kimbi J, Sumbele I, Ngufor L, Nadege K, Kimbi H. The epidemiological status of urogenital schistosomiasis among reproductive aged individuals in the Tiko Health Area- a semi-urban setting in the Mount Cameroon area. PLoS Negl Trop Dis. 2021;15. doi:10.1371/journal.pntd.0008978

73. Ndukwe YE, Obiezue RNN, Aguzie ION, Anunobi JT, Okafor FC. Mapping of Urinary Schistosomiasis in Anambra State, Nigeria. Ann Glob Health. 2019;85: 52. doi:10.5334/aogh.2393

74. Nigo M, Odermatt P, Salieb-Beugelaar G, Morozov O, Battegay M, Hunziker P. Epidemiology of Schistosoma mansoni infection in Ituri Province, north-eastern Democratic Republic of the Congo. PLoS Negl Trop Dis. 2021;15. doi:10.1371/journal.pntd.0009486

75. Njunda AL, Ndzi EN, Assob JCN, Kamga HLF, Kwenti ET. Prevalence and factors associated with urogenital schistosomiasis among primary school children in barrage, Magba sub-division of Cameroon. BMC Public Health. 2017;17. doi:10.1186/s12889-017-4539-6

76. Nzenou CGD, Tientche B, Asaah S, Takemegni WJM, Kenne M. An Update on Schistosomiasis: Prevalence, Intensity of Infection and Risk Factors among School-Aged Children in Njombe, Littoral Region, Cameroon. 2020;41.

77. Olamiju F, Nebe OJ, Mogaji H, Marcus A, Amodu-Agbi P, Urude RO, et al. Schistosomiasis outbreak during COVID-19 pandemic in Takum, northeast Nigeria: analysis of infection status and associated risk factors. PLoS ONE. 2022;17. doi:10.1371/journal.pone.0262524

78. Olkeba, Beekam Kebede, Boets P, Seid Tiku Mereta, Belayhun Mandefro, Gemechu Debesa, Mahmud Ahmednur, et al. Malacological and parasitological surveys on Ethiopian Rift Valley lakes: implications for control and elimination of snail-borne diseases. Int J Environ Res Public Health. 2021;19. doi:10.3390/ijerph19010142

79. Phiri BBW, Ngwira B, Kazembe LN. Analysing risk factors of co-occurrence of schistosomiasis haematobium and hookworm using bivariate regression models: case study of Chikwawa, Malawi. Parasite Epidemiol Control. 2016;1: 149–158. doi:10.1016/j.parepi.2016.02.001

80. Raja’a Y, Sulaiman S, Mubarak J, El-Bakri M, Al-Adimi W, El-Nabihi M, et al. Some aspects in the control of schistosomosis and soil-transmitted helminthosis in Yemeni children. SAUDI Med J. 2001;22: 428–432.

81. Raja’a Y, Assiragi H, Abu Luhom A, Mohammed A, Albahr M, Ashaddadi M, et al. Schistosomes infection rate in relation to environmental factors in school children. SAUDI Med J. 2000;21: 635–638.

82. Rollemberg C, Silva M, Rollemberg K, Amorim F, Lessa N, Santos M, et al. Predicting frequency distribution and influence of sociodemographic and behavioral risk factors of Schistosoma mansoni infection and analysis of co-infection with intestinal parasites. GEOSPATIAL Health. 2015;10: 13–19. doi:10.4081/gh.2015.303

83. Rudge JW, Stothard JR, Basáñez M-G, Mgeni AF, Khamis IS, Khamis AN, et al. Micro-epidemiology of urinary schistosomiasis in Zanzibar: Local risk factors associated with distribution of infections among schoolchildren and relevance for control. Acta Trop. 2008;105: 45–54. doi:10.1016/j.actatropica.2007.09.006

84. Ruganuza D, Mazigo H, Waihenya R, Morona D, Mkoji G. Schistosoma mansoni among pre-school children in Musozi village, Ukerewe Island, North-Western-Tanzania: prevalence and associated risk factors. Parasit Vectors. 2015;8. doi:10.1186/s13071-015-0997-9

85. Saad M, Mohamed S, Hosam EN, Hala E, Doaa S, Alaa G. Why schistosomiasis mansoni is still highly prevalent in rural EZBAS in Kafr El Sheikh, Egypt? Benha Med J. 2004;21: 305–330.

86. Salawu OT, Odaibo AB. Schistosomiasis transmission; socio-demographic, knowledge and practices as transmission risk factors in pregnant women. J Parasit Dis. 2016;40: 93–99. doi:10.1007/s12639-014-0454-2

87. Stothard J, Ameri H, Khamis I, Blair L, Nyandindi U, Kane R, et al. Parasitological and malacological surveys reveal urogenital schistosomiasis on Mafia Island, Tanzania to be an imported infection. ACTA Trop. 2013;128: 326–333. doi:10.1016/j.actatropica.2012.09.006

88. Sumbele IUN, Tabi DB, Teh RN, Njunda AL. Urogenital schistosomiasis burden in school-aged children in Tiko, Cameroon: a cross-sectional study on prevalence, intensity, knowledge and risk factors. Trop Med Health. 2021;49. doi:10.1186/s41182-021-00362-8

89. Takeuchi R, Njenga S, Ichinose Y, Kaneko S, Estrada C, Kobayashi J. Is there a gap between health education content and practice toward schistosomiasis prevention among schoolchildren along the shores of Lake Victoria in Kenya? PLoS Negl Trop Dis. 2019;13. doi:10.1371/journal.pntd.0007572

90. Umar S, Shinkafi SH, Hudu SA, Vasanthakumari Neela, Kumar Suresh, Syafinaz Amin Nordin, et al. Prevalence and molecular characterisation of Schistosoma haematobium among primary school children in Kebbi State, Nigeria. Ann Parasitol. 2017;63: 133–139.

91. Utzinger J, N’Goran EK, Tanner M, Lengeler C. Simple anamnestic questions and recalled water-contact patterns for self-diagnosis of Schistosoma mansoni infection among schoolchildren in western Cote d’Ivoire. Am J Trop Med Hyg. 2000;62: 649–655.

92. Wagatsuma Y, Aryeetey ME, Nkrumah FK, Sack DA, Kojima S. Highly symptom-aware children were heavily infected with urinary schistosomiasis in southern Ghana. Cent Afr J Med. 2003;49: 16–9.

93. Wanjala PM, Khaemba BM, Luoba AI. Prevalence and intensity of infection of intestinal Schistosomiasis and reinfection after intervention in Budalangi endemic focus of Western Kenya. Int J Trop Med. 2013;8: 71–80.

94. Wepnje G, Anchang-Kimbi J, Ndassi V, Lehman L, Kimbi H. Schistosoma haematobium infection status and its associated risk factors among pregnant women in Munyenge, South West Region, Cameroon following scale-up of communal piped water sources from 2014 to 2017: a cross-sectional study. BMC PUBLIC Health. 2019;19. doi:10.1186/s12889-019-6659-7

95. Wu ZW, Bu KM, Yuan LP, Yang GF, Zhu JH, Liu QL. Factors contributing to reinfection with schistosomiasis japonica after treatment in the lake region of China. Acta Trop. 1993;54: 83–88. doi:10.1016/0001-706X(93)90053-E

96. Wubet, Ketemaw, Damtie, Destaw. Prevalence of Schistosoma mansoni infection and associated risk factors among school children in Jiga town, northwest-Ethiopia: a cross-sectional study. J Parasitol Res. 2020;2020. doi:10.1155/2020/6903912

97. Ximenes RA de A, Southgate B, Smith PG, Guimaraes Neto L. Social environment, behavior, and schistosomiasis in an urban population in the northeast of Brazil. Rev Panam Salud Pública. 2001;9: 13–22.

98. Xu J, Xu J, Li S, Jia T, Huang X, Zhang H, et al. Transmission Risks of Schistosomiasis Japonica: Extraction from Back-propagation Artificial Neural Network and Logistic Regression Model. PLoS Negl Trop Dis. 2013;7. doi:10.1371/journal.pntd.0002123

99. Yacoub A, Southgate BA. The epidemiology of schistosomiasis in the later stages of a control programme based on chemotherapy: the Basrah study. 1. Descriptive epidemiology and parasitological results. Trans R Soc Trop Med Hyg. 1987;81: 449–459. doi:10.1016/0035-9203(87)90165-9

100. Zhou H, Ross A, Hartel G, Sleigh A, Williams G, McManus D, et al. Diagnosis of schistosomiasis japonica in Chinese schoolchildren by administration of a questionnaire. Trans R Soc Trop Med Hyg. 1998;92: 245–250. doi:10.1016/S0035-9203(98)90997-X

101. Zida A, Briegel J, Kabre I, Sawadogo M, Sangare I, Bamba S, et al. Epidemiological and clinical aspects of urogenital schistosomiasis in women, in Burkina Faso, West Africa. Infect Dis POVERTY. 2016;5. doi:10.1186/s40249-016-0174-1
