# Supplementary material for: Associations of water contact frequency, duration, and activities with schistosome infection risk: A systematic review and meta-analysis
Source: PLoS Negl Trop Dis. 2023 Jun 14;17(6):e0011377. doi: 10.1371/journal.pntd.0011377 (PMC10266691; doi:10.1371/journal.pntd.0011377)
Supplement: S3 Table — (DOCX) [file pntd.0011377.s015.docx]

## **S3 Table. Overview of final grouping of the exposure categories: having any water contact, water contact frequency water contact duration and water contact activities**

| **Exposure dimension** | **Exposure group** | **Exposure categories part of this group** | **Reference group** |
| --- | --- | --- | --- |
| Any water contact | Water contact | Water contact | No water contact |
| Frequency | Daily water contact | Daily water contact, daily or weekly water contact, water contact 1 to 2 times per day, water contact >7 times per week, water contact 7 times per week, water contact >3 times per day, water contact 3 times per day, water contact 2 times per day | No water contact/frequency of water contact<than frequency in exposure category |
|  | Weekly water contact | Weekly water contact, water contact 1 time per week, water contact 2-4 times per week, water contact >4 time per week, 8+ contacts over 2 weeks, water contact 3-6 times per week, water contact 1-2 times per week, >weekly water contact, 1 water contact in past week, >1 water contact in past week, water contact 3+ times per week, water contact 1-3 times per week, water contact 4-7 times per week, water contact more than 1 time per week | No water contact/frequency of water contact<than frequency in exposure category |
|  | Monthly water contact | 1-7 water contacts over 2 weeks, water contact twice a month or less, water contact 5-10 days per month, monthly water contact, 10-120 water contacts per year, >120 water contacts per year, water contact 1 time per week to 1 time per month | No water contact/frequency of water contact<than frequency in exposure category |
| Duration | Duration of water contact <= 1 hour | Water contact duration of activities >5 mins, water contact duration less than 10 minutes, water contact duration 10-60 minutes, 1-60 minutes of water contact, 5-15 mins of water contact per week | No water contact/duration of water contact<than duration in exposure category |
|  | Duration of water contact > 1 hour | Water contact duration more than than 60 minutes, water contact ≥3,445 minutes, water contact duration more than than 60 minutes, water contact duration 1-2 hours (per exposure), water contact duration >2 hours (per exposure) | No water contact/duration of water contact<than duration in exposure category |
| Activities | Domestic water contact activities | Getting water, washing, washing animals, washing clothes, laundry, watering garden at home, washing clothes or dishes, washing clothes daily, washing blankets, drinking open water | No water contact activity/not water contact exposure activity |
|  | Recreational water contact activities | Swimming/bathing, playing/bathing, swimming, playing, playing/swimming, swimming/playing, recreational water contact, swimming/fishing, swimming/playing/fishing with nets | No water contact activity/not water contact exposure activity |
|  | Occupational water contact activities | fishing/working, agriculture, irrigation, farming, fishing, sand extraction, cleaning streams, rice farming, farming/irrigation, fishing with rods, removal of vegetation, reed cutting, herding | No water contact activity/not water contact exposure activity |
