# Supplementary material for: Associations of water contact frequency, duration, and activities with schistosome infection risk: A systematic review and meta-analysis
Source: PLoS Negl Trop Dis. 2023 Jun 14;17(6):e0011377. doi: 10.1371/journal.pntd.0011377 (PMC10266691; doi:10.1371/journal.pntd.0011377)
Supplement: S2 Text — (DOCX) [file pntd.0011377.s018.docx]

# **S2 Text. Search strategy**

Logic of the search string is as follows (categories connected by ‘AND’):

| **Disease** | **Exposure** | **Outcome** |
| --- | --- | --- |
| (Schistosomiasis OR schistosom* OR bilharzi* OR "snail fever") | (water AND (contact* or pattern* or duration* or frequenc* or behavio* or exposure* or expose*)) | (risk or infection* or intensity or transmi* or odds or likelihood) |

The generic search string was adapted to suit constraints and functionalities of different databases. If possible, animal studies were excluded. Medline, Embase and Global Health were searched through Ovid (<https://www.wolterskluwer.com/en/solutions/ovid>). Web of Science was accessed through Clarivate ([https://clarivate.com](https://clarivate.com/)).

Interpretation of Medical Subject Headings (MeSH) terms used in search string:

- **Schistosomiasis**: "schistosomiasis"[MeSH Terms] OR "schistosomiasis"[All Fields] OR "schistosomiases"[All Fields]
- **water**: "water"[MeSH Terms] OR "water"[All Fields] OR "drinking water"[MeSH Terms] OR ("drinking"[All Fields] AND "water"[All Fields]) OR "drinking water"[All Fields] OR "watering"[All Fields] OR "water's"[All Fields] OR "watered"[All Fields] OR "waterer"[All Fields] OR "waterers"[All Fields] OR "waterings"[All Fields] OR "waters"[All Fields]
- **risk**: "risk"[MeSH Terms] OR "risk"[All Fields]
- intensity: "intense"[All Fields] OR "intensely"[All Fields] OR "intensities"[All Fields] OR "intensity"[All Fields] OR "intensively"[All Fields]
- **likelihood**: "likelihoods"[All Fields] OR "probability"[MeSH Terms] OR "probability"[All Fields] OR "likelihood"[All Fields]

Searches and search results (run 2022-05-13) for each database below:

**Medline (Ovid MEDLINE®) 1946 to present**

| **#** | **Search terms** | **Results** |
| --- | --- | --- |
| **1** | (Schistosomiasis or schistosom* or bilharzi* or "snail fever").mp. | 36713 |
| **2** | (water adj5 (contact* or pattern* or duration* or frequenc* or behavio* or exposure* or expose*)).mp. | 40244 |
| **3** | (risk or infection* or intensity or transmi* or odds or likelihood).mp. | 5843633 |
| **4** | 1 and 2 and 3 | 665 |
| **5** | exp Schistosomiasis/ep, tm [Epidemiology, Transmission] | 6188 |
| **6** | 2 and 5 | 487 |
| **7** | 4 or 6 | 681 |
| **8** | exp animals/ not humans.sh. | 5007245 |
| **9** | 7 not 8 | 639 |

[mp=title, abstract, original title, name of substance word, subject heading word, floating sub-heading word, keyword heading word, organism supplementary concept word, protocol supplementary concept word, rare disease supplementary concept word, unique identifier, synonyms]

[exp= explode search term to search for original term and all more specific terms nested within the original term]

[exp animals/ not humans.sh.=excludes animal studies]

[adj5=terms must co-occur within five words of each other]

**Embase 1974 to present**

| **#** | **Search terms** | **Results** |
| --- | --- | --- |
| **1** | (Schistosomiasis or schistosom* or bilharzi* or "snail fever").mp. | 38116 |
| **2** | (water adj5 (contact* or pattern* or duration* or frequenc* or behavio* or exposure* or expose*)).mp. | 44878 |
| **3** | (risk or infection* or intensity or transmi* or odds or likelihood).mp. | 7979480 |
| **4** | 1 and 2 and 3 | 790 |
| **5** | exp *schistosomiasis/ep [Epidemiology] | 2571 |
| **6** | 2 and 5 | 234 |
| **7** | 4 or 6 | 798 |
| **8** | (exp animals/ or nonhuman/) not human/ | 6953778 |
| **9** | 7 not 8 | 691 |
| **10** | conference*.pt. | 5169622 |
| **11** | 9 not 10 | 589 |

[conference*.pt.= search and exclude conference abstracts]

**Global Health 1973 to present**

| **#** | **Search terms** | **Results** |
| --- | --- | --- |
| **1** | (Schistosomiasis or schistosom* or bilharzi* or "snail fever").mp. | 35595 |
| **2** | (water adj5 (contact* or pattern* or duration* or frequenc* or behavio* or exposure* or expose*)).mp. | 11503 |
| **3** | (risk or infection* or intensity or transmi* or odds or likelihood).mp. | 1779612 |
| **4** | 1 and 2 and 3 | 926 |
| **5** | exp Schistosomiasis | 30955 |
| **6** | 2 and 5 | 897 |
| **7** | 4 or 6 | 926 |

**Global Index Medicus 1901 to present**

| **#** | **Search terms** | **Results** |
| --- | --- | --- |
| **1** | tw:(Schistosomiasis or schistosom* or bilharzi* or "snail fever") | 3772 |
| **2** | tw:(water AND (contact* or pattern* or duration* or frequenc* or behavio* or exposure* or expose*)) | 11905 |
| **3** | tw: (risk or infection* or intensity or transmi* or odds or likelihood) | 360100 |
| **4** | 1 and 2 and 3 | 282 |

[tw=title,abstract,subheading]

**Web of Science – Core Collection, Science Citation Index Expanded 1900 to present**

| **#** | **Search terms** | **Results** |
| --- | --- | --- |
| **1** | ALL=(Schistosomiasis or schistosom* or bilharzi* or "snail fever") | [34,593](https://www.webofscience.com/wos/woscc/summary/211ff979-546d-466b-b822-2a23cb94916e-38984f95/date-descending/1) |
| **2** | AB=(water NEAR/5 (contact* or pattern* or duration* or frequenc* or behavio* or exposure* or expose*)) | [107,655](https://www.webofscience.com/wos/woscc/summary/f4e9af21-2cc9-4a23-a57f-61137f762e80-33c047a6/date-descending/1) |
| **3** | ALL=(risk or infection* or intensity or transmi* or odds or likelihood) | [6,752,340](https://www.webofscience.com/wos/woscc/summary/91b8d558-a9db-4b0f-a41c-78ba2a261c59-33c057b4/date-descending/1) |
| **4** | 1 and 2 and 3 | [458](https://www.webofscience.com/wos/woscc/summary/7bed5928-3f59-4abd-b2af-650c89d49a4f-33c05faf/date-descending/1) |
| **5** | TS=(animals or animal or mice or mus or mouse or murine or woodmouse or rats or rat or murinae or muridae or cottonrat or cottonrats or hamster or hamsters or cricetinae or rodentia or rodent or rodents or pigs or pig or swine or swines or piglets or piglet or boar or boars or sus scrofa or ferrets or ferret or polecat or polecats or mustela putorius or guinea pigs or guinea pig or cavia or callithrix or marmoset or marmosets or cebuella or hapale or octodon or chinchilla or chinchillas or gerbillinae or gerbil or gerbils or jird or jirds or merione or meriones or rabbits or rabbit or hares or hare or diptera or flies or fly or dipteral or drosphila or drosophilidae or cats or cat or carus or felis or nematoda or nematode or nematoda or nematode or nematodes or sipunculida or dogs or dog or canine or canines or canis or sheep or sheeps or mouflon or mouflons or ovis or goats or goat or capra or capras or rupicapra or chamois or haplorhini or monkey or monkeys or anthropoidea or anthropoids or saguinus or tamarin or tamarins or leontopithecus or hominidae or ape or apes or pan or paniscus or pan paniscus or bonobo or bonobos or troglodytes or pan troglodytes or gibbon or gibbons or siamang or siamangs or nomascus or symphalangus or chimpanzee or chimpanzees or prosimians or bush baby or prosimian or bush babies or galagos or galago or pongidae or gorilla or gorillas or pongo or pygmaeus or pongo pygmaeus or orangutans or pygmaeus or lemur or lemurs or lemuridae or horse or horses or pongo or equus or cow or calf or bull or chicken or chickens or gallus or quail or bird or birds or quails or poultry or poultries or fowl or fowls or reptile or reptilia or reptiles or snakes or snake or lizard or lizards or alligator or alligators or crocodile or crocodiles or turtle or turtles or amphibian or amphibians or amphibia or frog or frogs or bombina or salientia or toad or toads or epidalea calamita or salamander or salamanders or eel or eels or fish or fishes or pisces or catfish or catfishes or siluriformes or arius or heteropneustes or sheatfish or perch or perches or percidae or perca or trout or trouts or char or chars or salvelinus or fathead minnow or minnow or cyprinidae or carps or carp or zebrafish or zebrafishes or goldfish or goldfishes or guppy or guppies or chub or chubs or tinca or barbels or barbus or pimephales or promelas or poecilia reticulata or mullet or mullets or seahorse or seahorses or mugil curema or atlantic cod or shark or sharks or catshark or anguilla or salmonid or salmonids or whitefish or whitefishes or salmon or salmons or sole or solea or sea lamprey or lamprey or lampreys or pumpkinseed or sunfish or sunfishes or tilapia or tilapias or turbot or turbots or flatfish or flatfishes or sciuridae or squirrel or squirrels or chipmunk or chipmunks or suslik or susliks or vole or voles or lemming or lemmings or muskrat or muskrats or lemmus or otter or otters or marten or martens or martes or weasel or badger or badgers or ermine or mink or minks or sable or sables or gulo or gulos or wolverine or wolverines or minks or mustela or llama or llamas or alpaca or alpacas or camelid or camelids or guanaco or guanacos or chiroptera or chiropteras or bat or bats or fox or foxes or iguana or iguanas or xenopus laevis or parakeet or parakeets or parrot or parrots or donkey or donkeys or mule or mules or zebra or zebras or shrew or shrews or bison or bisons or buffalo or buffaloes or deer or deers or bear or bears or panda or pandas or wild hog or wild boar or fitchew or fitch or beaver or beavers or jerboa or jerboas or capybara or capybaras) | [7,527,847](https://www.webofscience.com/wos/woscc/summary/a9477e8c-23c0-4691-978d-308d90e09cb8-33c08fbd/date-descending/1) |
| **6** | 4 not 5 | [343](https://www.webofscience.com/wos/woscc/summary/4dfbc1ea-a48d-4453-bb4a-088d83b01e81-38986e65/date-descending/1) |

[ALL=all terms, TS=Topic, AB=abstract]

[NEAR/5=terms must co-occur within five words of each other]

[term #5 excludes animal studies]

**Cochrane Central Register of Controlled Trials (CENTRAL) 1996 to present**

| **#** | **Search terms** | **Results** |
| --- | --- | --- |
| **1** | (Schistosomiasis or schistosom* or bilharzi* or "snail fever") | 744 |
| **2** | (water AND (contact* or pattern* or duration* or frequenc* or behavio* or exposure* or expose*)) | 212 |
| **3** | (risk or infection* or intensity or transmi* or odds or likelihood) | 455761 |
| **4** | 1 and 2 and 3 | 46 |

[title, abstract, keywords were searched for terms]

**Interpretation of MeSH terms:**

- **Schistosomiasis**: "schistosomiasis"[MeSH Terms] OR "schistosomiasis"[All Fields] OR "schistosomiases"[All Fields]
- **water**: "water"[MeSH Terms] OR "water"[All Fields] OR "drinking water"[MeSH Terms] OR ("drinking"[All Fields] AND "water"[All Fields]) OR "drinking water"[All Fields] OR "watering"[All Fields] OR "water's"[All Fields] OR "watered"[All Fields] OR "waterer"[All Fields] OR "waterers"[All Fields] OR "waterings"[All Fields] OR "waters"[All Fields]
- **risk**: "risk"[MeSH Terms] OR "risk"[All Fields]
- intensity: "intense"[All Fields] OR "intensely"[All Fields] OR "intensities"[All Fields] OR "intensity"[All Fields] OR "intensively"[All Fields]
- **likelihood**: "likelihoods"[All Fields] OR "probability"[MeSH Terms] OR "probability"[All Fields] OR "likelihood"[All Fields]
