# Supplementary material for: Associations of water contact frequency, duration, and activities with schistosome infection risk: A systematic review and meta-analysis
Source: PLoS Negl Trop Dis. 2023 Jun 14;17(6):e0011377. doi: 10.1371/journal.pntd.0011377 (PMC10266691; doi:10.1371/journal.pntd.0011377)
Supplement: S3 Text — (DOCX) [file pntd.0011377.s019.docx]

# **S3 Text. Notes on data extraction**

1. Age: we classified age as follows: PSAC (0-4), SACs (5-14), older children and adults (15+). Studies were classified as all age categories they intersected with. For instance, studies with a population aged 10-45 would be classified as SACs/older children and adults.
2. GPS coordinates were extracted from study area information containing information on the study villages/schools/health centre and higher-level administrative areas in which the study location was nested. GPS coordinates were assigned as follows:
   1. If the study was conducted in one village/school/health centre, the precise GPS were extracted from Google Maps
   2. If the study was conducted across multiple villages/schools/health centres, the midpoint of those locations was extracted from Google Maps
   3. If no villages/schools/health centre was identifiable, the midpoint of the higher administrative area was extracted from Google Maps
   4. If the study was a country-wide representative study, the midpoint of the country was extracted from Google Maps
3. Locality: urban/rural/peri-urban were assigned as reported by the study authors.
4. Water setting: if the statistical analysis reported a specific water setting (e.g. OR of water contact with lake), this setting was assigned. Otherwise, all water settings reported in the description of the study area were listed as water settings. When water contact was reported separately for multiple waterbodies (e.g. OR of water contact with lake and OR of water contact with river) and we could not reconstruct an overall measure of water contact, contact with the largest water body was included.
5. Sample size: the analytical sample size of associations between water contact and infection was extracted (sometimes these associations were only reported on a subset of the larger study sample).
6. Effect sizes: we extracted all effect sizes for water contact (overall water contact, frequency, duration, or activities). We did not extract measures where the reference category was not provided or unclear. We extracted ORs wherever possible. Otherwise, we extracted raw participant numbers to populate 2x2 tables. We extracted all effect sizes for water contact measures that were provided as overall water contact, frequency, duration, or activities. We always used the entirety of data available to reconstruct data, including figures, tables, supplementary appendices, and datasets provided. We ran models based on data provided in supplements for two papers [1,2]. We used an automated graph extraction tool (graphreader, <http://www.graphreader.com>) to obtain data from one paper [3]. Two authors provided the 2x2 tables we requested [4,5].
7. Climate zone: we matched study GPS coordinates (which we obtained through the process described above), with Köppen-Geiger climate zone at the study location. We used gridded data providing a Köppen-Geiger climate zone classification for the period between 1980–2016 at a 1-km resolution [6] and assigned the modal climate zone based on a 25-km buffer around each study location. This buffer accounts for uncertainty about the precise study location as well as for uncertainty in studies covering larger geographic areas.
8. Safe drinking water: we extracted information on whether the study provided ORs adjusted for drinking water source.
9. Sanitation: we extracted information on whether the study provided ORs adjusted for whether participants had access to sanitation (private or public sanitation infrastructure).

**References**

1. Exum NG, Kibira SPS, Ssenyonga R, Nobili J, Shannon AK, Ssempebwa JC, et al. The prevalence of schistosomiasis in Uganda: A nationally representative population estimate to inform control programs and water and sanitation interventions. Akullian A, editor. PLoS Negl Trop Dis. 2019;13: e0007617. doi:10.1371/journal.pntd.0007617

2. Gazzinelli A, Oliveira-Prado R, Matoso L, Veloso B, Andrade G, Kloos H, et al. Schistosoma mansoni reinfection: Analysis of risk factors by classification and regression tree (CART) modeling. PLoS One. 2017;12. doi:10.1371/journal.pone.0182197

3. Butterworth AE, Dalton PR, Dunne DW, Mugambi M, Ouma JH, Richardson BA, et al. Immunity after treatment of human schistosomiasis mansoni. I. Study design, pretreatment observations and the results of treatment. Trans R Soc Trop Med Hyg. 1984;78: 108–123. doi:10.1016/0035-9203(84)90190-1

4. Moira AP de, Fulford AJC, Kabatereine NB, Ouma JH, Booth M, Dunne DW. Analysis of complex patterns of human exposure and immunity to schistosomiasis mansoni: the influence of age, sex, ethnicity and IgE. PLoS Negl Trop Dis. 2010;4: e820. doi:10.1371/journal.pntd.0000820

5. Atalabi TE, Lawal U, Akinluyi FO. Urogenital schistosomiasis and associated determinant factors among senior high school students in the Dutsin-Ma and Safana Local Government Areas of Katsina State, Nigeria. Infect Dis Poverty. 2016;5.

6. Beck HE, Zimmermann NE, McVicar TR, Vergopolan N, Berg A, Wood EF. Present and future Köppen-Geiger climate classification maps at 1-km resolution. Sci Data. 2018;5: 180214. doi:10.1038/sdata.2018.214
