# Supplementary material for: Associations of water contact frequency, duration, and activities with schistosome infection risk: A systematic review and meta-analysis
Source: PLoS Negl Trop Dis. 2023 Jun 14;17(6):e0011377. doi: 10.1371/journal.pntd.0011377 (PMC10266691; doi:10.1371/journal.pntd.0011377)
Supplement: S4 Text — (DOCX) [file pntd.0011377.s020.docx]

# **S4 Text. Quality appraisal tool**

We used a modified version of the Quality Assessment Tool for Observational Cohort and Cross-Sectional Studies from the National Institutes of Health (NIH, Bethesda, MD, United States of America) to perform the quality appraisal [1]. The tool was applied to all eligible study designs: cohort studies, cross-sectional studies, case-control studies, before-after studies (if reporting on baseline infection and exposure) and randomised controlled trials (if reporting on infection and exposure at baseline or in the control group).

Studies were graded as ‘yes/no/unclear’ on each question. The overall score was derived as follows: the number of questions graded as ‘yes’ translated into one point each, whereas questions scored as ‘no’ or ‘unclear’ (i.e. when studies failed to report relevant information) were scored with zero points. Possible overall scores ranged from 0-11. We graded studies with studies with scores of 0-3 as ‘low quality’, studies with a score of 4-7 as ‘intermediate quality’ and studies with an overall score of 8-11 as ‘high quality’.

## **Quality assessment categories**

| **Category** | **Question** | **Select 'yes' if** | **Select ‘no’ if** |
| --- | --- | --- | --- |
| Aim | Was the research question or objective in this paper clearly stated? | Authors clearly describe goal of their research and state the population, exposure and outcome, e.g. the aim was to estimate the agreement between lake water contact by direct observation every 3 months and self-reported water contact in community members aged 5+ and to establish its relationship with *S. mansoni* infection | No aim or unspecific aim, e.g. this study examines the water contact behaviour of an isolated farm-worker community |
| Representativeness | Was the study population clearly specified and defined? Were inclusion and exclusion criteria for being in the study prespecified and applied uniformly to all participants? (For case-control studies, were cases and controls clearly differentiated?) | Authors describe the group of people from which the study participants were selected or recruited, using demographics, location, and time period; e.g. school-age children, male and female (aged 5-14), enrolled on Oct 1, 2014, in the five schools in the catchment areas of study villages were eligible | Description lacking specifics on study population demographics, location, and time period, e.g. community members living in five communities were the study population |
| Representativeness | Is the sampling method clearly described? Is the sample representative of the population from which it is drawn and was a sample size justification, power description, or variance and effect estimates provided? (For RCTs was the method of randomisation described and is risk of bias low? For case-control studies, was selection of cases and controls clearly described?) | Sampling method clearly described, e.g. ten school-age children (5-14) per class in each of the five schools were sampled using stratified random sampling, statistical power was assessed | Lacking details to understand how sampling was done, e.g. 50 adults per village were selected for inclusion in the study, no justification for sample size provided |
| Representativeness | Response rate >50% or differences between respondents and non-respondents described? Was loss to follow-up after baseline 20% or less (if applicable)? | Provides statistics on response rate, e.g. 93% of respondents selected from village registries agreed to participate in the study, or differences in known characteristics of respondents and non-respondents (age, gender) were assessed using t-tests, loss to follow-up was 15% (if applicable) | Lacking information on response rate and differences between respondents and non-respondents provided |
| Exposure | Was water contact clearly defined, valid (e.g. covering daytime hours), reliable, and implemented consistently across all study participants? | Observation/recall measure of water contact includes all daytime hours, e.g. measure of typical river water contact per week, direct water contact observation from 6am-6pm | Recall/observation limited to specific daytime period, e.g. direct observation of water contact from 12pm-3pm |
| Exposure | Is the sought level of detail (spatial and temporal) feasible with the chosen measurement method and adequate for establishing water contact? | Level of detail in recall is realistic, e.g. self-reported water contact frequency over past week, direct water contact observation reported in minutes; level of detail is sufficient to establish water contact, e.g. logger interval of 2 minutes | Unrealistic level of detail, e.g. self-reported frequency of water contact over past month (unrealistically long recall), self-reported duration of average water contact (unrealistic level of precision); insufficient detail to establish water contact, e.g. logger interval of 30 minutes, mobile phone traces with coarse spatial resolution |
| Exposure | Did the study examine different levels (i.e. more than two levels) of water contact as related to the outcome? | Study describes variable construction and clearly defines three or more different levels of water contact and defines unit of measurement, e.g. 0-1 times per week, 2-5 time per week, more than 5 times per week | Study constructs binary variable of water contact or doesn't define variable construction and measurement unit, e.g. water contact/no water contact |
| Outcomes | Outcome measurement was done by trained personnel, using two samples/readings of same sample (if microscopy method), using validated commercial test (if POC-CCA or PCR) | Clearly states number of samples/readings (for microscopy) or provides evidence for reliability of diagnostic test, e.g. two readings of slides were done independently by two trained technicians; POC-CCA test validated by assessing agreement between POC-CCA and microscopy or reference to studies which demonstrate accuracy of specific test | Lacks detail on microscopy, e.g. Kato-Katz examination was used to determine infection status; no evidence of reliability of diagnostic test provided |
| Outcomes | Were exposures and outcomes measured independently by two people? | Water contact measurement and diagnostic test done by different people, e.g. self-reported water contact using survey (exposure) and microscopy by a trained technician (outcome) | Same person measuring water contact and infection, e.g. technicians responsible for microscopy (outcome measurement) recruited to contact direct water contact observation (exposure) |
| Analysis | Were adjusted effect estimates shown? | Studies report both types of models, e.g. unadjusted association between any water contact in past 7 days and infection status when compared to no water contact was OR = 1.49, 95% CI 1.12, 1.97, adjusted association was OR = 1.23, 95% CI 0.98, 1.48 | Studies report only adjusted or unadjusted ORs/RRs, fail to provide 95% CIs |
| Analysis | Do adjusted estimates account for age and gender? | Studies report the covariates adjusted for, e.g. OR between any water contact in past 7 days and infection status OR = 1.49 [95% CI 1.12; 1.97], when adjusting for age and gender | Studies fail to provide descriptions of variables adjusted for, e.g. adjusted OR for infection for respondents with any water contact compared to no water contact was OR = 1.49 (no further info on adjustment available) |

**References**

1. National Heart, Lung, and Blood Institute. Observational Cohort and Cross-Sectional Studies from the National Institutes of Health. Available: https://www.nhlbi.nih.gov/health-topics/study-quality-assessment-tools
