# Supplementary material for: Associations of water contact frequency, duration, and activities with schistosome infection risk: A systematic review and meta-analysis
Source: PLoS Negl Trop Dis. 2023 Jun 14;17(6):e0011377. doi: 10.1371/journal.pntd.0011377 (PMC10266691; doi:10.1371/journal.pntd.0011377)
Supplement: Attachment — Submitted filename: PLOS_review.docx [file pntd.0011377.s021.docx]

**Associations of water contact frequency, duration, and activities with schistosome**

**infection risk: A systematic review and meta-analysis**

This is a systematic review and meta-analysis focused on understanding the relationship between water sources contacts and the risk of schistosomes transmission in endemic areas. The paper addresses a complex issue but present a common knowledge of water contacts and transmission of schistosomes. The role of WASH is given priority and more evidence on the need of WASH to be integrated in the MDA. The paper is worth to be published after minor corrections.

**Abstract**

44-47:- If 10 out of 101 studies stated water contact measurements as their study aims (9.9%), the remaining studies were 91(91%) and not 97%.

- What about the Schistosome species in different water sources?
- What about snails’ abundance? water contacts only without infected snails may not determine the transmission of Schistosomes. Can it come as limitation.
- In the model- can you run fishing and agriculture activities as separate variables
- Limitation:- data from fishing population especially adult are very few

**Background**

- Is too long
- Line 235-237- What about urine filtration?-if the study included also S.haematobium
- Line 237-338-What about the size of water bodies? Does it have effect on transmission? Any influence on snails’ abundance and transmission intensity?
- What about seasonal water bodies Vs permanent water bodies? Consider that there are water bodies which dries completely during the dry season and the transmission goes to zero.

**Discussion**

- Line 507-diagnostic tests used? Add the word test

- In general, the section is too long and readers looses focus though it focuses on the results of the analysis.

- 661-665- Not sure if authors consider that in the same areas with seasonal water bodies, during the dry season, most of the water bodies dries out and transmission of Schistosomes goes to zero until the next rain season.
