# Supplementary material for: Associations of water contact frequency, duration, and activities with schistosome infection risk: A systematic review and meta-analysis
Source: PLoS Negl Trop Dis. 2023 Jun 14;17(6):e0011377. doi: 10.1371/journal.pntd.0011377 (PMC10266691; doi:10.1371/journal.pntd.0011377)
Supplement: Attachment — Submitted filename: Response_to_reviewers_20230505.docx [file pntd.0011377.s022.docx]

**RESPONSE TO REVIEWER COMMENTS**

**REVIEWER 1**

**Reviewer 1, comment 1:** The methods are well explained and robust. The results have been analysed appropriately and presented clearly.

*Response*: We thank the reviewer for acknowledging the strengths of the manuscript and for providing useful feedback for the interpretation of the results.

**Reviewer 1, comment 2:** The conclusions are supported by the data presented. Just one suggested minor addition is that more could be said in the Discussion section about the need to re-define 'WASH' when we talk about schistosomiasis prevention, both in terms of the types of 'WASH' infrastructure that are needed and the level of community access and coverage that must be achieved. Typically, 'WASH' refers to clean drinking water, toilets and handsoap. Indeed, universal access and usage to these components of WASH would eventually have a significant impact on schistosomiasis prevalence. However, the results of the study suggest that even small amounts of water contact can maintain infection levels, meaning that, at least in the near term, there is an urgent need to provide water infrastructure to facilitate safe water contact activities and replace the usage of contaminated water bodies for such activities. Examples may include safe laundry basins, showering/bathing facilities, or swimming facilities. It should be emphasised that when we say 'WASH' for schistosomiasis prevention, the 'W' must include enough safe water for all of a community's water contact needs, not just for drinking water. This is an under-emphasised point and means that even when communities are provided with traditional 'WASH' at sub-universal access levels, there is likely to still be significant schistosomiasis transmission risk.

*Response:* We found this comment useful as it helps us to more fully spell out the implications of this review for the WASH community. In lines 659-668 of the discussion section, we have added: “In terms of WASH, our results suggest that achieving a critical threshold of (near-)universal community-level coverage may be needed for WASH to successfully control transmission. As almost any water contact activity was associated with infection risk, lowering community-level exposure and contamination may require not just access to safe drinking water, but providing a whole suite of facilities including laundry basins, showering or bathing facilities, toilets, or swimming facilities that minimise all types of open water contact. We were unable to directly assess how access to safe water or sanitation modified individual-level risk of water contact using subgroup analysis as none of the 33 studies in our main analysis provided effect sizes adjusted by access to safe drinking water or sanitation.”

To further investigate the role of WASH in modifying associations between having any water contact and infection, we also extracted additional data from the studies to understand whether studies adjusted for participants access to safe water or sanitation in their analysis. Among 101 studies, only three studies adjusted for drinking water access and one study adjusted for sanitation infrastructure. Due to these very low numbers, we were not able to conduct a subgroup analysis of water contact and infection between studies who adjusted for WASH to studies who did not.

**REVIEWER 2**

**Reviewer 2, comment 1**: Line 44-47: If 10 out of 101 studies stated water contact measurements as their study aims (9.9%), the remaining studies were 91(91%) and not 97%.

*Response:* Thank you for this clarification. We streamlined and slightly shortened the abstract and took out the sentence you referred to. Lines 44-46 now read as follows: “Included studies mostly reported on water contact activities (69%; 70/101) and having any water contact (33%; 33/101). Ninety-six percent of studies (97/101) used surveys to measure exposure”.

**Reviewer 2, comment 2:** What about the Schistosome species in different water sources?

*Response:* Thank you for this comment. Given that both schistosome and snail species may vary across different waterbodies, we provide two tables here to address your question. For all the waterbodies included in the subgroup analysis in Figure 5 (lakes, streams, rivers), we report on the distribution of schistosome and snail species.

In terms of schistosome species, we find that *S. haematobium* was more frequently studied in research that focused on rivers or streams and *S. mansoni* was mostly the focus of studies which took place along lakes. We had added this finding in lines 400-403. This is very likely driven by the geographic variation of schistosome species. For instance, the Great Lakes are in East Africa where *S. mansoni* is abundant and the *S. japonicum* studies are from the Poyang Lake Region in China, hence the relatively more frequent occurrence of *S. japonicum* and *S. mansoni* in studies with lakes.

We also added schistosome species as an additional subgroup in Figure 5 and report on it in lines 398-400. We find that schistosome species present (*S. haematobium* versus *S. mansoni*) did not significantly modify infection likelihood.

| **Waterbody type** | **n** | ***S mansoni* (%)** | ***S. haematobium* (%)** | ***S. japonicum* (%)** | ***Multiple species* (%)** |
| --- | --- | --- | --- | --- | --- |
| river | 58 | 0.379 | 0.500 | 0.017 | 0.103 |
| stream | 35 | 0.286 | 0.600 | 0.000 | 0.114 |
| lake | 24 | 0.542 | 0.292 | 0.125 | 0.042 |
| **Overall** | 101 | 0.416 | 0.465 | 0.050 | 0.069 |

In terms of snail species broken down by waterbody type, we observe the following distribution:

| **Waterbody type** | **n** | **Snail species not reported (%)** | **Snail species reported (%)** | ***Biomphalaria spp.* (%)** | ***Bulinus spp.* (%)** | ***Lymnaea spp.* (%)** | ***Oncomelania spp.* (%)** |
| --- | --- | --- | --- | --- | --- | --- | --- |
| river | 58 | 0.517 | 0.483 | 0.310 | 0.224 | 0.017 | 0.017 |
| stream | 35 | 0.343 | 0.657 | 0.343 | 0.371 | 0.000 | 0.000 |
| lake | 24 | 0.625 | 0.375 | 0.250 | 0.167 | 0.042 | 0.083 |
| **Overall** | 101 | 0.535 | 0.465 | 0.267 | 0.228 | 0.010 | 0.020 |

Whereas all studies reported on the schistosome species studied, the genus or species of snail intermediate host was infrequently shared. Information on snails was shared in less than half of all studies (46.5%, information could be either be from malacology or by referring to other research). Overall, 26.7% of studies were conducted in areas where snails of a *Biomphalaria spp.* were present and 22.8% of studies were in areas where snails of *Biomphalaria spp* were present. Other species, such as *Oncomelania spp* and *Lymnaea spp.* were rarely reported. There were no clear differences in the snail species studied by waterbody type. *Biomphalaria spp.* tended to be slightly more frequently present in rivers and lakes compared to *Bulinus spp.* *Bulinus spp* were slightly more frequent in studies that focused on streams. Note, each study often had multiple water body types further complicating the separation of intermediate hosts.

**Reviewer 2, comment 3:** What about snails’ abundance? water contacts only without infected snails may not determine the transmission of Schistosomes. Can it come as limitation.

*Response*: Thank you for this remark. Abundance of infected snails is an important determinant of environmental risk. To address your comment, we were aiming to conduct a subgroup analysis that compared associations of having any water contact and infection in studies that found infected snails with those studies that did not find infected snails. However, abundance of snails and prevalence of infected snails were not commonly reported in studies. Among 101 studies, only 21.8% reported on snail abundance (from malacology) and 12.8% reported on prevalence of infected snails. In order to study snail variables with water contact, we need to have snail data reported for each comparable measure of water contact exposure. For example, among the 33 studies in our main analysis (any water contact) for which we aimed to conduct a subgroup analysis based on abundance, we had four studies that included malacology (three which reported snails were abundant and one which found no snails). These numbers were too low to conduct a subgroup meta-analysis. We added this as a limitation of the studies available in lines 701-706. Your comment identifies an important gap in the literature which is the need to jointly model the human and environmental factors that determine infection likelihood.

**Reviewer 2, comment 4:** In the model- can you run fishing and agriculture activities as separate variables Limitation:- data from fishing population especially adult are very few.

Thank you for this comment. To clarify, the results we show in Figure 8, Panel (A) are from separate models which report on associations between farming and fishing with schistosome infection. The forest plot displays the results from separate models in a single figure to allow for easy comparison of the results across the separate models. Our analysis shows that engaging in fishing was associated with 2.38 times higher odds of infection compared to not fishing. Farming was associated with 3.02 times higher odds of infection compared to not engaging in farming. A limitation of the analysis is that activity categories were often not clearly defined, for instance, irrigation may refer to irrigated agriculture or it may also refer to irrigation for domestic purposes, such as watering plants. Therefore, we decided to not group farming and irrigation together since the definitions across studies did not allow us to conclude that irrigation was always for the purposes of farming.

The point you make on fishing in adult populations is an important one. We focus on fishing as an activity (which could be conducted by study participant of any age). We also extracted information of fishing as an occupation which is from adult fishing populations but only 3 out of 101 studies reported on infection risk based on fishing as an occupation (as reported in lines 630-631). Therefore, we could only report on fishing as an activity, but not as an occupation.

**Reviewer 2, comment 5:** Background is too long.

*Response:* We have made some edits in the background section to shorten it.

**Reviewer 2, comment 6:** Line 235-237- What about urine filtration?- if the study included also *S. haematobium*.

*Response:* Thank you for noting this typo. We do not restrict by diagnostic in this meta-analysis and have revised the text. In lines 250-253, it now reads: “Studies were grouped by endemicity setting following the 2022 World Health Organization (WHO) guideline definitions for mass drug administration (MDA) treatment (low < 10% prevalence, moderate = 10-49% prevalence, high ≥ 50% prevalence)”.

**Reviewer 2, comment 7:** Line 237-338-What about the size of water bodies? Does it have effect on transmission? Any influence on snails’ abundance and transmission intensity?

*Response*: Waterbody size is an important environmental factor that may influence transmission because of its influence on snail dynamics and human behaviour. In the context of a systematic review, there is no robust way to directly extract information on waterbody size from the included studies. An indirect way to understand the influence of waterbody size is by interpreting the coefficients in Figure 5 of the subgroup analysis by waterbody type in light of waterbody size. The results show that amongst the included waterbodies (streams, rivers, lakes), streams which are the smallest water body here have the largest effect size. To investigate the effect of waterbody size on transmission, future studies should explore the use of remote sensing as a novel tool to obtain objective measures of waterbody size.

**Reviewer 2, comment 8:** What about seasonal water bodies Vs permanent water bodies? Consider that there are water bodies which dries completely during the dry season and the transmission goes to zero.

*Response*: As highlighted in lines 717-719, past research has often found that transmission is more intense during the dry season. You make an important point in saying that if water bodies completely dry out, transmission may be lower (or even zero). We tried to investigate this by extracting information on whether studies were conducted during the dry season. Among 101 studies, 14.8% reported on dry season water contact. Yet, among the 33 studies in our main analysis, only three reported on dry season water contact. By conducting a new subgroup meta-analysis based on dry season, we find that the association between having any water contact and infection likelihood is lower in studies conducted in the dry season (2.41 [0.87; 6.63], n=3 studies) compared to studies not conducted in the dry season (OR 3.22 [95% CI 2.10; 4.95], n=30 studies). However, since the subgroup with the dry season was extremely small (only three studies), we do not think that this additional analysis provides dependable evidence; the results also are not statistically different. We believe that using climate zone as a proxy provides for a more objective way to capture climatic differences as this classification method relies on geographic location which was available in similar quality across all studies. In lines 720-723, we then also reported on the frequency with which different waterbodies were studied within these different climate zones to link climate and waterbody characteristics. Our post-hoc analysis which relied on in-text information about dry-season water contact means that we cannot distinguish between studies conducted in locations where there is a dry season (but the study was conducted in the rainy season) and studies conducted in locations where there is no dry season. Therefore, we believe that climate zone information is a more dependable measure for whether there is a dry season in a given study location. There is a need to better understand how infection risk changes across seasons and which waterbody characteristics are responsible for variation in seasonal transmission risk.

**Reviewer 2, comment 9:** Line 507-diagnostic tests used? Add the word test.

*Response:* Thank you for catching this. We have added the missing word.

**Reviewer 2, comment 10:** In general, the section is too long and readers looses focus though it focuses on the results of the analysis.

*Response:* Thank you for this comment. We have added subject sub-headings in the discussion section to make it easier for the reader to follow.

**Reviewer 2, comment 11:** 661-665- Not sure if authors consider that in the same areas with seasonal water bodies, during the dry season, most of the water bodies dries out and transmission of Schistosomes goes to zero until the next rain season.

*Response*: Thank you for this comment. This is closely related to our response to comment 8. Any statements on the effect of the dry season on water contact are limited by the very low number of dry-season studies here. We do find that in our subgroup of studies (n=3) conducted in the dry season there is still a positive, albeit insignificant association between water contact and infection (OR 2.41 [0.87; 6.63]). However, what makes this question difficult to answer in the context of our review are inherent selection biases in water contact studies. It is very unlikely that researchers would decide to conduct a water contact study in a setting where all waterbodies have dried out.
